# c-Maf Deletion in Cortical Somatostatin, But Not Parvalbumin, Interneurons Leads to Absence-Like Epileptiform Activity in Mice

**DOI:** 10.1523/ENEURO.0257-25.2026

**Published:** 2026-07-17

**Authors:** Morgane A. Leroux, Jia Sheng Hu, Yuliya Voskobiynyk, Emily Ling-Lin Pai, Irene Lew, Marie Burkart, John L. R. Rubenstein, Jeanne T. Paz

**Affiliations:** ^1^Gladstone Institute of Neurological Disease, Gladstone Institutes, San Francisco, California 94158; ^2^The Kavli Institute for Fundamental Neuroscience and Neurology Department, University of California San Francisco, San Francisco, California 94158; ^3^Institut des Systèmes Intelligents et de Robotique (ISIR), Sorbonne University, Paris 75005, France; ^4^Brain-Cognition-Behavior Graduate Program, Sorbonne University, Paris 75005, France; ^5^Department of Psychiatry, University of California San Francisco, San Francisco, California 94158; ^6^Neuroscience Graduate Program, University of California, San Francisco, California 94158

**Keywords:** absence epilepsy, cortical development, electrophysiology, interneurons, spike and wave discharges

## Abstract

Mafb and c-Maf transcription factors (TFs) are expressed in medial ganglionic eminence (MGE) lineages, beginning in progenitors and continuing into mature GABAergic parvalbumin-positive (PV^+^) and somatostatin-positive (SST^+^) cortical interneurons (CINs). Deleting Mafb and c-Maf in MGE before SST versus PV fate specification causes developmental anomalies, including altered numbers of CINs and seizure phenotypes, but the specific contributions of these TFs in postmitotic SST^+^ and PV^+^ CINs to epilepsy remain unknown. To address this, we conditionally deleted Mafb or c-Maf in SST^+^ or PV^+^ interneurons after interneuron fate specification in female and male mice. Deletion of c-Maf, but not Mafb, in SST+ cells was associated with reduced synaptic excitation onto these cells and with spontaneous spike-and-wave discharges, consistent with absence-like seizures. In contrast, deletion of Mafb in SST^+^ CINs reduced their density in superficial cortical layers but did not induce epilepsy. Neither c-Maf nor Mafb deletion in PV^+^ CINs produced major electrophysiological or histological abnormalities in the somatosensory cortex. These findings identify a specific requirement for c-Maf in modulating synaptic excitation of SST^+^ interneurons and show that its loss in SST^+^ cells is associated with the development of absence-like epileptiform activity in vivo. Together, our results refine the understanding of how transcriptional programs shape interneuron function in the mature cortex and highlight c-Maf/MAF-dependent pathways as candidates for investigation in epilepsy genetics.

## Graphical abstract

**Figure:**
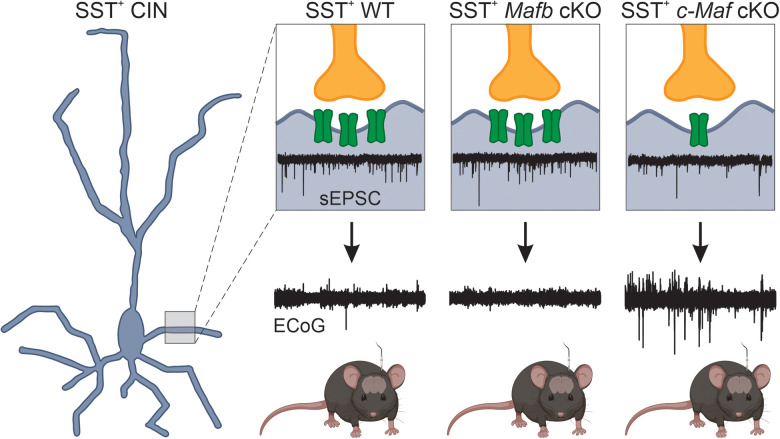
Visual Abstract

## Significance Statement

Disruptions in cortical circuit development and function are linked to neurodevelopmental disorders. Cortical interneurons (CINs), particularly the two major subtypes—somatostatin-positive (SST^+^) and parvalbumin-positive (PV^+^) cells—help maintain excitation/inhibition balance. Although Mafb and c-Maf transcription factors are known to regulate CIN development, their roles in mature SST^+^ and PV^+^ cells remain unclear. We show that conditional deletion of c-Maf in postmitotic SST^+^ interneurons alters their synaptic excitation and is associated with the development of spontaneous epileptiform activity in mice. Deletion of either gene in PV^+^ cells had no such effect. These findings reveal a specific vulnerability of SST^+^ cells to c-Maf loss and underscore the need to investigate c-MAF as a potential contributor to epilepsy and related disorders in humans.

## Introduction

A number of phenotypes observed in epilepsy and autism spectrum disorder are thought to reflect disturbances in the development and function of the neocortex. In particular, imbalance in the excitation/inhibition of cortical circuits may underlie aspects of these disorders (Rubenstein and Merzenich, 2003; Chao et al., 2010; Yizhar et al., 2011; Han et al., 2012). The excitation/inhibition balance is controlled in part by GABAergic cortical interneurons (CINs). Thus, defects in CINs may underlie some neurological and neuropsychiatric disorders.

CINs originate from subpallial telencephalic regions called the medial ganglionic eminence (MGE) and the caudal ganglionic eminence (Anderson et al., 1997; Marín and Rubenstein, 2001; Nery et al., 2002). CINs have diverse morphological, connectivity, molecular, and electrophysiological properties (Huang et al., 2007; Kepecs and Fishell, 2014; Kessaris et al., 2014). Mature CINs that are derived from the MGE express distinct molecular markers, such as somatostatin (SST^+^) and parvalbumin (PV^+^; Lim et al., 2018). SST^+^ and PV^+^ CINs are GABAergic and represent the majority of CINs, regulate the excitability of pyramidal neurons, and maintain excitation/inhibition balance (Markram et al., 2004; Rudy et al., 2011). SST^+^ and PV^+^ CINs have distinct functions. For instance, PV^+^ CINs control the vertical spread of excitability in cortical layers, whereas SST^+^ CINs control the horizontal spread (Markram et al., 2004; DeFelipe et al., 2013). These complementary actions provide versatile and potent control of the excitability of neocortical circuits. SST^+^ interneurons have been shown to play critical roles in regulating network excitability during epileptiform activity, including contributing to feedforward inhibition and limiting the propagation of ictal-like discharges (Parrish et al., 2019; Wengert et al., 2021; Drexel et al., 2022). Although PV^+^ interneuron dysfunction is often implicated in epilepsy, whether selective cell-autonomous genetic disruption of SST^+^ interneurons alone can lead to spontaneous absence-like network epileptiform activity remains less clear.

The development and function of SST^+^ and PV^+^ CINs are known to be regulated by a combination of transcription factors (TFs) that include the *Arx*, *Dlx1/2/5/6*, *Lhx6/8*, *Mafb/c*, *Mef2c*, *Nkx2-1*, *Npas1/3*, *Nr2f1/2*, *Satb1*, and *Zeb2* gene families (Wonders and Anderson, 2006; Gelman et al., 2011; Hu et al., 2017; Lim et al., 2018; Pla et al., 2018; Pai et al., 2020). Removal of either *c-Maf* or *Mafb*—basic leucine-zipper TFs—in the MGE and its lineages using *Nkx2.1-Cre* and *799-CreER* has been shown to lead to alterations in the electrical properties of SST^+^ and PV^+^ CINs (Pai et al., 2019) and to electrographic seizures (Pai et al., 2020). However, deleting these genes at such an early developmental stage also led to significant developmental anomalies, such as a reduction in the number of CINs or alterations in their fates (Pai et al., 2019, 2020), making it challenging to dissect the role of *Maf* genes across different types of CINs. As a result, the roles of *Mafb* and *c-Maf* genes after the specification of CIN fates (SST^+^ or PV^+^) have so far remained unknown.

To address this gap, we conditionally deleted *Mafb* or *c-Maf* in either SST^+^ or PV^+^ CINs using mice expressing either *Somatostatin-IRES-Cre* (Taniguchi et al., 2011) or *Parvalbumin-Cre* (Hippenmeyer et al., 2005). We then analyzed the impact of these deletions on electrocorticographic activity in adult mice and on the electrophysiological properties of adult PV^+^ and SST^+^ CINs in mouse brain slices.

## Materials and Methods

### Contact for reagents and resource sharing

Requests for further information, resources, and reagents should be directed to and will be fulfilled by the lead contacts.

### Experimental model

All protocols were approved by the Institutional Animal Care and Use Committee. Precautions were taken to minimize stress and the number of animals used in each set of experiments. Mice were separately housed after surgical implants.

The following mouse strains have been previously reported: *tdTomato^lox/+^* (Ai14) Cre reporter (Madisen et al., 2010), *Maf(c-Maf)^lox/+^* (Wende et al., 2012), *Mafb^lox/+^* (Yu et al., 2013), *Somatostatin-IRES-Cre* (Taniguchi et al., 2011), and *Parvalbumin-Cre* (Hippenmeyer et al., 2005). All strains were on a mixed C57BL/6 and CD1 background. All animals were housed in a vivarium with a 12/12 h light/dark cycle. Mice used for experiments were housed with their littermates. Both males and females were used in the experiments.

The *Maf ^lox^* allele (on chromosome 8) contains *loxP* sites 1,547 bp upstream of and 411 bp into the open reading frame (i.e., outside of and within the gene's lone exon). The *Mafb ^lox^* allele (on chromosome 2) contains *loxP* sites upstream of the gene's lone exon and at the end of the exon. Recombination caused by *Somatostatin-IRES-Cre* begins at embryonic day 12.5 (E12.5). Recombination caused by *Parvalbumin-Cre* begins at postnatal day 10 (P10). The conditional knock-out mice will be referred to as cKO hereafter.

### Immunohistochemistry

Mice were anesthetized with intraperitoneal avertin (0.015 ml/g of a 2.5% solution) and perfused transcardially with PBS followed by 4% PFA. Brains were isolated and fixed with PFA for synapse immunohistochemistry (IHC) or in situ hybridization (ISH). After 2 d of cryoprotection, the brains were sectioned with a microtome (40-µm-thick coronal sections).

For all IHC on postnatal brains, staining was carried out on free-floating sections. For synapse IHC, sections were pretreated with pepsin for 10 min at 37°C to enhance staining and then incubated with primary antibodies for 2 d. Immunofluorescent labeling was performed with the following primary antibodies: mouse calretinin (1:250, Swant, 6B3), rabbit nNOS (1:200, Life Technologies, 61-7000), rabbit parvalbumin (1:300, Swant, PV25), mouse PSD95 (1:500, NeuroMab, 75-028 clone ID K28/43), and rabbit Vglut1 (1:500, Synaptic Systems, 135303). We performed all IHC on *n* ≥ 3 biological replicates, meaning at least three brains from each genotype.

### ISH

We performed ISH on a minimum of *n* = 3 biological replicates (i.e., three brains per genotype) for each control and mutant. In each case, a rostrocaudal series of at least 10 sections was examined. ISHs were performed using digoxigenin-labeled riboprobes.

### Image acquisition and processing

Fluorescent IHC images in Figure 3 were taken using a Coolsnap camera (Photometrics) mounted on a Nikon Eclipse 80i microscope using NIS-Elements acquisition software (Nikon). Bright-field ISH images in Figure 3 were taken using a DP70 camera (Olympus) mounted on an Olympus SZX7 microscope. Brightness and contrast were adjusted, and images were merged using the ImageJ software. Synapse IHC images from neocortical layers II/III were taken on an OMX-SR confocal microscope with a 60× objective at 1,024 × 1,024 pixels of resolution. Synapse IHC images were then aligned and deconvolved before synapse counting in Extended Data Figure 5-1.

### Cell and synapse counting

For assessing cell densities, 10× images were taken in the primary somatosensory cortex (S1) at postnatal ages from two or three nonadjacent sections and from both hemispheres for each replicate. A box of defined area was drawn over a region of interest. Cells were counted within that box and were divided by the box area. Cell density was quantified in a blinded manner. DAPI was used to distinguish neocortical layers.

For synapse counting, confocal image stacks (0.25 µm step size) were processed with ImageJ. Background subtraction was applied, and vGlut1 and PSD95 channels were merged. We counted the number of synapses colocalizing with vGlut1 and PSD95 in each focal plane. Synapse numbers were then normalized to the length of the dendrite. The length of the dendrite was determined by the segmented line that was drawn over the dendrite where synapses were counted. Synapse density was quantified in a blinded manner. Ten total dendrites from two replicates were quantified per genotype (Extended Data Fig. 5-1).

### Slice preparation for electrophysiology

Mice were killed with 4% isoflurane, perfused with ice-cold sucrose cutting solution containing (in mM) 234 sucrose, 2.5 KCl, 1.25 NaH_2_PO_4_, 10 MgSO_4_, 0.5 CaCl_2_, 26 NaHCO_3_, and 11 glucose, equilibrated with 95% O_2_ and 5% CO_2_, pH 7.4, and decapitated. We prepared 250-µm-thick horizontal thalamic slices containing the somatosensory barrel cortex with a Leica VT1200 microtome (Leica Microsystems). We incubated the slices, initially at 32°C for 1 h and then at 24–26°C, in artificial cerebrospinal fluid containing (in mM) 126 NaCl, 2.5 KCl, 1.25 NaH_2_PO_4_, 2 MgCl_2_, 2 CaCl_2_, 26 NaHCO_3_, and 10 glucose, equilibrated with 95% O2 and 5% CO2, pH 7.4.

### Whole-cell patch–clamp electrophysiology

We visually identified the interneurons by their tdTomato expression. Neurons were identified by differential contrast optics with a Zeiss (Oberkochen) Axioskop microscope and an infrared video camera. Recording electrodes made of borosilicate glass had a resistance of 2.5–4 MΩ when filled with intracellular solution. Access resistance was monitored in all the recordings, and cells were included for analysis only if the access resistance was <25 MΩ. The access resistance was similar in all SST^+^ and PV^+^ neurons (WT, *Mafb* cKO, and *c-Maf* cKO; *p* > 0.5), indicating comparable recording quality across genotypes. Spontaneous excitatory postsynaptic currents (sEPSCs) were recorded in the presence of picrotoxin (50 µM, Tocris Bioscience). For sEPSCs and current-clamp recordings, the internal solution contained (in mM) 120 potassium gluconate, 11 KCl, 1 MgCl2, 1 CaCl2, 10 HEPES, and 1 EGTA, pH adjusted to 7.4 with KOH (290 mOsm). The experiments were performed by blinded observers.

### Surgical implantation of devices for electrocorticographic recordings in freely behaving mice

The devices for electrocorticography (ECoG) recordings in freely behaving mice were made using a Mill-Max base (ED90267-ND, Mill-Max) to collect electrical activity from multiple cortical regions simultaneously, as described in Ritter-Makinson et al. (2019). Each device contained multiple screws for ECoG acquisition. ECoG screws were implanted bilaterally in the S1 cortex (S1, at −0.5 mm posterior to the bregma, ±3.25 mm lateral), in the prefrontal cortex (PFC, at +1.78 mm anterior to the bregma, ±0 mm lateral), and in the right primary visual cortex (V1, at −2.9 mm posterior to the bregma, +3.25 mm lateral). A ground screw was placed over the cerebellum (1 mm posterior and 0.5 mm lateral to the lambda). Mice were allowed to recover for at least 1 week before recording. ECoG signals were recorded using RZ5 (Tucker Davis Technologies, TDT) and sampled at 1,221 Hz. A video camera synchronized to the signal acquisition was used to monitor the animals during the recordings. Animals were briefly anesthetized with ∼2% isoflurane at the start of each recording session to connect the animal to the recording equipment. Each recording trial lasted 60–180 min. To control for circadian rhythms, we housed our animals under a regular light/dark cycle and recorded between 9:00 A.M. and 6:00 P.M.

### Quantification and statistical analysis

Complete statistical reporting is provided in Extended Data Figure 1-1. Bar graphs show mean ± SEM unless otherwise indicated. Statistical analyses were performed using GraphPad Prism (version 7) and R (version 4.6.0), and a *p* value of <0.05 was considered significant. The specific *n* for each experiment can be found in the Results section, figures, legends, and Extended Data Figure 1-1. Data from male and female mice were included in the analysis.

#### Spontaneous epileptiform spike-and-wave discharges

*Terminology*. Throughout the manuscript, we use the following terms consistently. A spike-and-wave discharge (SWD) is a single electrographic event lasting 1–5 s, composed of rhythmic spike-and-wave complexes (SWCs). A SWC is the individual spike-and-wave cycle within an SWD; the dominant within-discharge frequency (∼8 Hz) reflects the recurrence rate of SWCs. The epileptiform spike is the sharp spike component of each complex, detected as described below and quantified as epileptiform spikes per hour. We use absence-like epileptiform activity as the overarching descriptive term for the SWD phenotype, which was accompanied by behavioral arrest, and reserve absence-like seizures for this behaviorally associated activity, by analogy to rodent models of absence epilepsy.

The dominant frequency of the ECoG during the SWDs was calculated by fast Fourier transforms using the Power Spectrum tool in Spike2 (version 7.20, Cambridge Electronic Design). Analysis of the SWCs in ECoG was performed using Spike2 (version 7.20, Cambridge Electronic Design; Holden et al., 2021). The frequency of SWCs was quantified by detecting the epileptiform spike component (defined as a sharp event exceeding seven times the root mean square of the baseline ECoG; Holden et al., 2021). All detected events were visually validated by a scientist blinded to the groups. Statistical analysis was carried out on the GraphPad Prism software (version 9.2.0) using Kruskal–Wallis one-way ANOVA followed by Dunn's multiple-comparisons test.

#### SST^+^ and PV^+^ cell densities

For all cell and synapse counts from IHC and ISH images, we used the Cell Counter plugin in FIJI. Statistical analyses were carried out in Python (scipy.stats, v3.10.12) and R (v4.6.0); linear mixed-effects models were fit using the lme4 and lmerTest packages following the RMeDPower2 framework (Shin et al., 2022). Normality was assessed using a Shapiro–Wilk test and homoscedasticity using a Levene's test. For SST^+^ and PV^+^ cell densities (Fig. 3, Extended Data Fig. 3-1), the all-layers (total) cell density per mouse was analyzed by one-way ANOVA followed by pairwise two-tailed Student's *t* tests with Holm–Šidák correction across the three pairwise genotype contrasts (*k* = 3). Per-layer cell density (Fig. 3 only) was additionally analyzed using linear mixed-effects regression (density ∼ Genotype + (1 | mouse_id)) at each cortical layer, with Holm–Šidák correction across all simple effects (3 pairwise genotype contrasts × 4 cortical layers; *k* = 12) within each panel. All Figure 3 and Extended Data Figure 3-1 panels passed parametric assumptions (Shapiro–Wilk *p* > 0.05 in every group; Levene *p* > 0.05).

#### SST^+^ and PV^+^ intrinsic and synaptic electrophysiological properties

Electrophysiological (Figs. 4, 5) and synaptic analyses (Extended Data Fig. 5-1) were performed at earlier ages than ECoG to assess cellular phenotypes independently of chronic network adaptations observed in older animals. Whole-cell patch–clamp recordings were analyzed using Clampfit (version 10.7). For each cell, we extracted the input–output relationship (F–I curve: spike count at each of nine 20 pA current steps from 20 to 180 pA), active membrane properties (action potential threshold, amplitude, half-width, and rheobase), passive membrane properties (resting membrane potential, input resistance, membrane time constant, and capacitance), and sEPSCs (sEPSC amplitude, decay tau, charge, and instantaneous frequency).

Linear mixed-effects models were fit by REML in R (v4.6) using the lme4 and lmerTest packages (Bates et al., 2015; Kuznetsova et al., 2017), with degrees of freedom and *p* values for fixed effects estimated via Satterthwaite's approximation, following the RMeDPower2 framework (Shin et al., 2022). Multiple-comparison corrections were applied using base R's p.adjust and a custom Holm–Šidák implementation; all three correction methods (Bonferroni, Holm–Šidák, Benjamini–Hochberg FDR) gave the same qualitative conclusions.

F–I curves were modeled by per-step linear mixed-effects regression. Spike count was reshaped from wide to long format (one observation per cell per current step). For each cell type and each current step from 20 to 180 pA in 20 pA increments, we fit spike_count ∼ genotype + (1 | mouse_id) by REML using lme4 and lmerTest, computed all three pairwise genotype contrasts as described above, and corrected within each contrast type across the nine current steps (*k* = 9) by Holm–Šidák. We additionally fit an omnibus interaction LMM, spike_count ∼ genotype * current_pA + (1 + current_pA | mouse_id:cell_id) + (1 | mouse_id), to test whether the slope of the F–I curve differs across genotypes; interaction terms were tested by Satterthwaite *t* tests. The 0 pA step (all cells silent) and currents above 180 pA (too few cells maintained sustained firing for reliable inference) were excluded from the analyzed range. Sex was not included as a covariate in the F–I models because the SST^+^ c-Maf cKO electrophysiology cohort included only female mice (Figs. 4, 5), and sensitivity analyses suggested that omission of sex did not alter the qualitative conclusions.

For all other electrophysiological properties, models were fit independently per measurement on cells with a non-NA value for that measurement (na_action: “unique”); modeled *n* equals the per-measure cell count given in the figure legends. Mouse counts per group were unchanged across measurements. For each cell type (PV^+^, SST^+^) and each measurement (sEPSC amplitude, decay tau, charge, instantaneous frequency; AP threshold, amplitude, half-width, rheobase; Vm, Rin, tau_m, Cm), we fit a linear mixed-effects model with genotype as a three-level fixed effect (WT, *Mafb* cKO, *c-Maf* cKO) and a random intercept for individual mouse to account for nonindependence of cells recorded from the same animal. Sex was included as an additional fixed-effect covariate for PV^+^ models only; it was omitted from SST^+^ models because the *c-Maf* cKO electrophysiology cohort contributed no males, rendering the sex term unidentifiable in that cell type. Across the eight PV^+^ intrinsic models, the sex coefficient (M vs F) reached the uncorrected 0.05 threshold for rheobase, with males requiring ∼25 pA less injected current than females (β = −25.0 pA, Satterthwaite *p* = 0.021); no other sex coefficient was significant in any sEPSC or intrinsic model. Dropping the sex term did not change any qualitative conclusion regarding genotype contrasts, but the term was retained because the sexes were imbalanced across genotypes within PV^+^ recordings.

For each model, we computed all three pairwise genotype contrasts: *Mafb* cKO versus WT, *c-Maf* cKO versus WT, and *c-Maf* cKO versus *Mafb* cKO. The parametrization in R places WT as the first factor level so that two of these contrasts appear directly as model coefficients; the third contrast was constructed as a linear combination of those coefficients with standard error derived from the fixed-effect variance-covariance matrix and Satterthwaite degrees of freedom (lmerTest::contest1D). All three contrasts and their standard errors are invariant to the choice of the reference level. Within each model, the three pairwise comparisons (*k* = 3) were corrected by the Holm–Šidák step-down method to control the familywise error rate; adjusted *p* values are reported. No correction was applied across measurements within a cell type or across the two cell types.

#### SST^+^ and PV^+^ synapse densities

Synaptic puncta density was quantified per dendrite as puncta per pixel along confocally imaged dendrites of SST^+^ CINs (Extended Data Fig. 5-1). We sampled 30 dendrites from six mice (10 dendrites and two mice per genotype). For all synapse counts from IHC, we used the Cell Counter plugin in FIJI.

Statistical analyses were carried out using R (version 4.6.0) for fitting linear mixed-effects models using the lme4 and lmerTest packages following the RMeDPower2 framework (Shin et al., 2022) and Python's (version 3.10.12) scipy.stats package. Because the mouse-level *n* was small, this exploratory analysis is reported at the dendrite level (where each dendrite is the unit of analysis); dendrite-level conclusions were cross-checked against a linear mixed-effects model with mouse as a random intercept and against a per-mouse one-way ANOVA on dendrite-averaged values, both of which yielded effects in the same direction but did not reach significance at the biological-replicate level (Extended Data Fig. 5-1*B*). The dendritic puncta-per-pixel distributions were positive, right-skewed, and bounded near zero and were therefore well approximated by lognormal distributions (verified by *Q*–*Q* plots of log-transformed values). For each genotype, we fit a lognormal distribution to the dendrite measurements by maximum likelihood. To test for between-genotype distributional differences, we performed pairwise likelihood-ratio tests against a pooled-fit null (*χ*^2^ with df = 2) and corrected for the three pairwise comparisons by Bonferroni’s method (*m* = 3). Empirical cumulative distributions and fitted lognormal CDFs are shown in Extended Data Figure 5-1.

## Results

### *Deletion of c-Maf*, but not of *Mafb*, in SST^+^ interneurons leads to SWDs in mice

**Figure 1.**
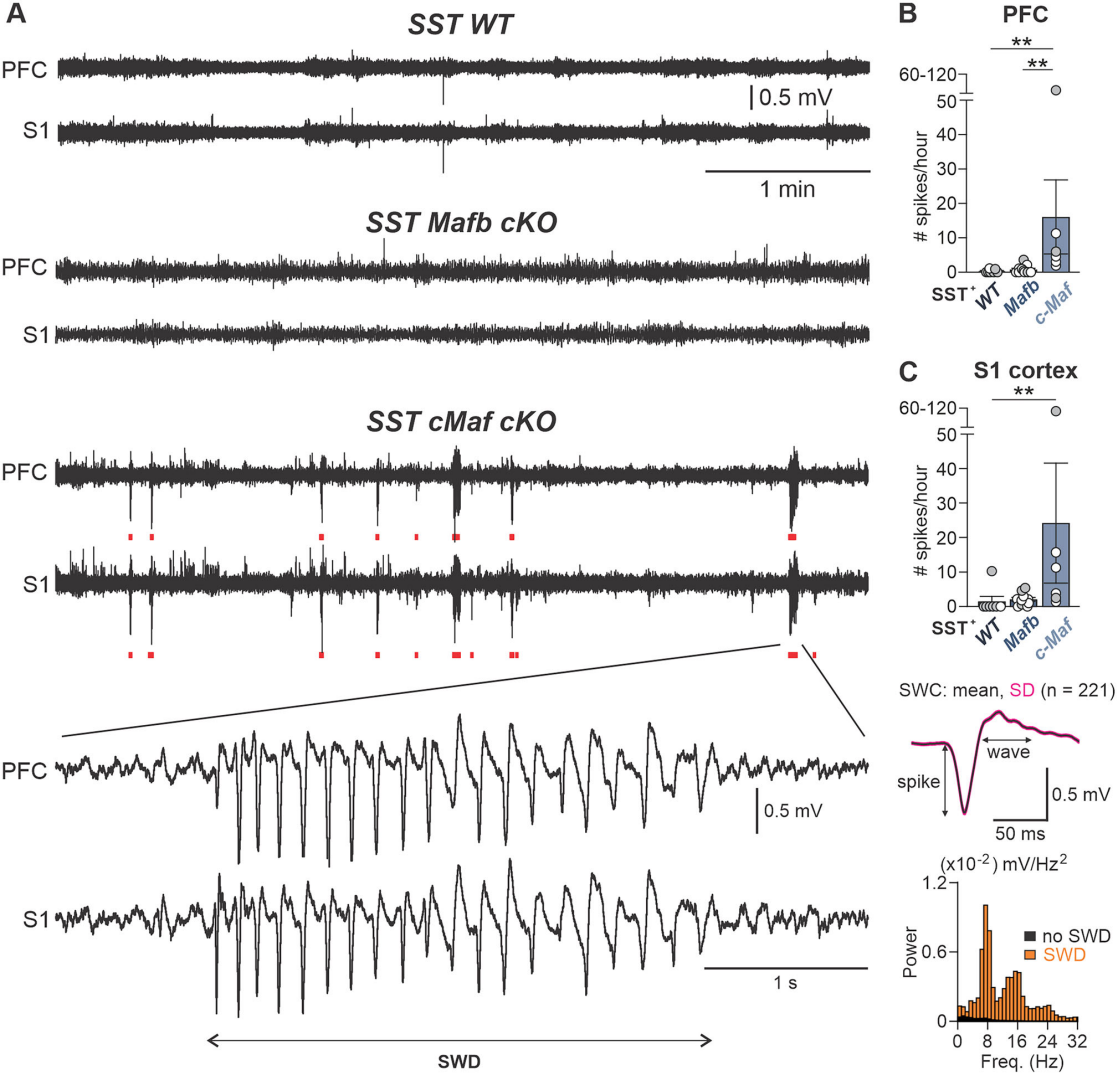
Deletion of c-Maf, but not Mafb, in SST^+^ interneurons leads to spontaneous epileptiform SWDs. ***A***, Representative ECoG recordings from the PFC and S1 cortex of WT, SST^+^ *Mafb* cKO, and SST^+^ *c-Maf* cKO mice (epileptiform spikes are indicated in red). Bottom left, Representative SWD. Bottom right, Averaged SWC (mean ± SD; *n* = 221 events from a single representative mouse) and overlaid fast Fourier transforms of SWDs (*n* = 20 SWDs, orange) and baseline ECoG activity recorded between SWDs (black) from the same mouse. Note the peak at 8 Hz and a harmonic at 16 Hz. ***B***, ***C***, The number of epileptiform spikes per hour recorded in the PFC (***B***) and S1 cortex (***C***). Data are presented as mean ± SEM. Statistical significance was assessed using a Kruskal–Wallis one-way ANOVA followed by Dunn's multiple-comparisons post hoc test (**p < 0.01). Males are represented by gray dots and females by white dots. ECoG recordings were performed in 8–9-month-old mice. WT, *n* = 7 mice; SST^+^ *Mafb* cKO, *n* = 10 mice; SST^+^ *c-Maf* cKO, *n* = 6 mice. See Extended Data Figure 1-1 for complete statistical reporting.

**Figure 2.**
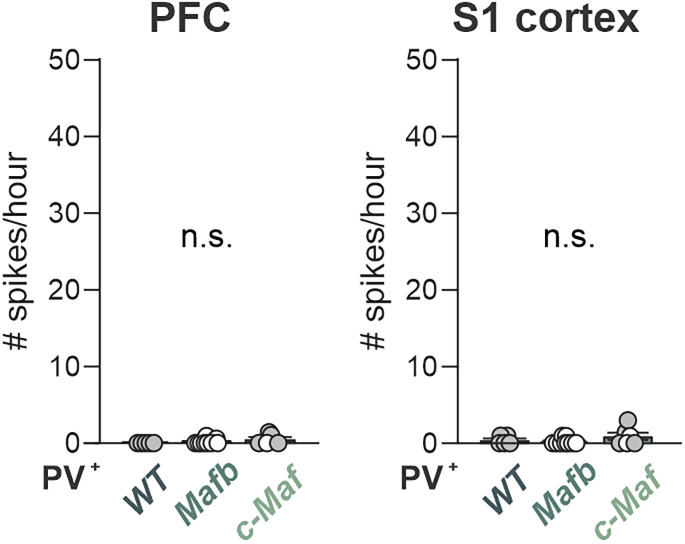
Deletion of Mafb or c-Maf in PV^+^ CINs does not alter SWDs in adult mice. ***A***, ***B***, Number of epileptiform spikes per hour detected in ECoG recordings in the PFC (***A***) or S1 cortex (***B***) from WT, PV^+^ *Mafb* cKO, and PV^+^ *c-Maf* cKO mice. Data are presented as mean ± SEM. Statistical significance was assessed using Kruskal–Wallis one-way ANOVA followed by Dunn's multiple-comparisons test (ns, not significant). Males are represented by gray dots and females by white dots. ECoG recordings were performed in 8–9-month-old mice. WT, *n* = 5 mice; PV^+^ *Mafb* cKO, *n* = 9 mice; PV^+^ *c-Maf* cKO, *n* = 6 mice (PFC *n* = 5 mice due to a noisy electrode in one mouse). See Extended Data Figure 1-1 for complete statistical reporting.

To determine if conditionally deleting *Mafb* or *c-Maf* in either SST^+^ or PV^+^ cells results in seizures, we crossed mice carrying floxed (loxP-flanked) alleles of these genes to mice expressing either *Somatostatin-IRES-Cre mice* (Taniguchi et al., 2011), which drives recombination beginning around E12.5, or *Parvalbumin-Cre* (Hippenmeyer et al., 2005), which drives recombination postnatally around P10. In both cases, Cre-mediated recombination occurs after SST^+^ and PV^+^ interneuron fate specification. We then implanted chronic ECoG devices into the PFC and the S1 cortex of (1) SST^+^ *Mafb* cKO, SST^+^ *c-Maf* cKO, and wild-type littermates (Fig. 1) and (2) PV^+^ *Mafb* cKO, PV^+^ *c-Maf* cKO, and wild-type littermates (Fig. 2). Video-ECoG was recorded for 2–4 h 3 weeks after surgery. These experiments were done on 8–10-month-old mice to match the age used in Pai et al. (2020) in which *Mafb* and *c-Maf* were deleted in MGE progenitors before CIN fate specification.

10.1523/ENEURO.0257-25.2026.f1-1 Figure 1-1 **Statistical Summary Table.** This table provides a comprehensive overview of the statistical analyses and descriptive metrics corresponding to the experimental data presented in Figures 1 through 5 (including extended data panels). Data are derived from wildtype (WT), *Mafb* conditional knockout (*Mafb* cKO), and *c-Maf* conditional knockout (*c-Maf* cKO) mouse lines within both Somatostatin-cre and Parvalbumin-cre cohorts of mice. Descriptive statistics are presented as mean ± SEM unless otherwise noted. **Abbreviations and definitions: *Genotypes and Cohorts:*** WT: Wildtype control; Mafb cKO: *Mafb* conditional knockout; c-Maf cKO: *c-Maf* conditional knockout; SST+: Somatostatin-expressing; PV+: Parvalbumin-expressing; CIN: Cortical interneuron. ***Anatomical and histological terms*:** PFC: Prefrontal cortex; S1: Primary somatosensory cortex; Layers II/III, IV, V, VI: Specific neocortical layers; ISH: In situ hybridization; tdTomato: Fluorescent reporter protein used for genetic cell lineage tracking; nNOS+: Neuronal nitric oxide synthase-expressing (marking long-range SST + neurons). ***Electrophysiological parameters*:** AP: Action potential; Vm: Resting membrane potential (measured in mV); Rin: Input resistance (measured in MΩ); τm (tau_m): Membrane time constant (measured in ms); Cm: Membrane capacitance (measured in pF); Rheobase: Minimum current required to elicit an action potential (measured in pA); sEPSC: Spontaneous excitatory postsynaptic current; Decay tau: Synaptic current decay time constant. Units of Measurement: mo: months of age; Hz: Hertz (cycles per second); pA: picoamperes; fC: femtocoulombs; ms / mV: milliseconds / millivolts. ***Statistical nomenclature and significance thresholds*:** n: sample size (explicitly indicated as number of mice, sections, or individual patched cells where appropriate); ANOVA: Analysis of variance (used for parametric group comparisons); Kruskal–Wallis (K–W): Non-parametric equivalent to one-way ANOVA used for non-normally distributed data; LMM: Linear mixed-effects model (used to account for nested data structures, such as multiple cells or sections recorded per individual mouse ID); LRT: Likelihood-ratio test; HSD: Honestly Significant Difference (Tukey's post-hoc test); k / m: Multiple-comparison correction factors (e.g., family-wise error adjustments via Holm–Šidák or Bonferroni methods); n.s.: Not significant (p > 0.05). ***Significance multipliers*:** Adjusted p-values are annotated in the table using a standard alpha-level star tier system: *p < 0.05; **p < 0.01; *** p < 0.001; **** p < 0.0001. Download Figure 1-1, DOCX file.

We found that SST^+^ *c-Maf* cKO but not *Mafb* cKO mice had SWDs in both the PFC (Fig. 1*A*) and the S1 cortex (Fig. 1*B*). These SWDs lasted 1–5 s, with SWCs peaking at 8 Hz, and were associated with behavioral arrest, consistent with absence epilepsy-like activity in rodent models (Paz et al., 2007; Sorokin et al., 2017). On the other hand, deletion of *Mafb* or *c-Maf* in PV^+^ CINs did not induce SWCs in the PFC (Fig. 2*A*) or in the S1 cortex (Fig. 2*B*). Thus, *c-Maf* deletion alone in SST^+^ cells results in spontaneous electrographic absence-like epileptiform activity under our experimental conditions.

### *c-Maf* deletion in SST^+^ interneurons does not affect SST^+^ CIN density in the neocortex

**Figure 3.**
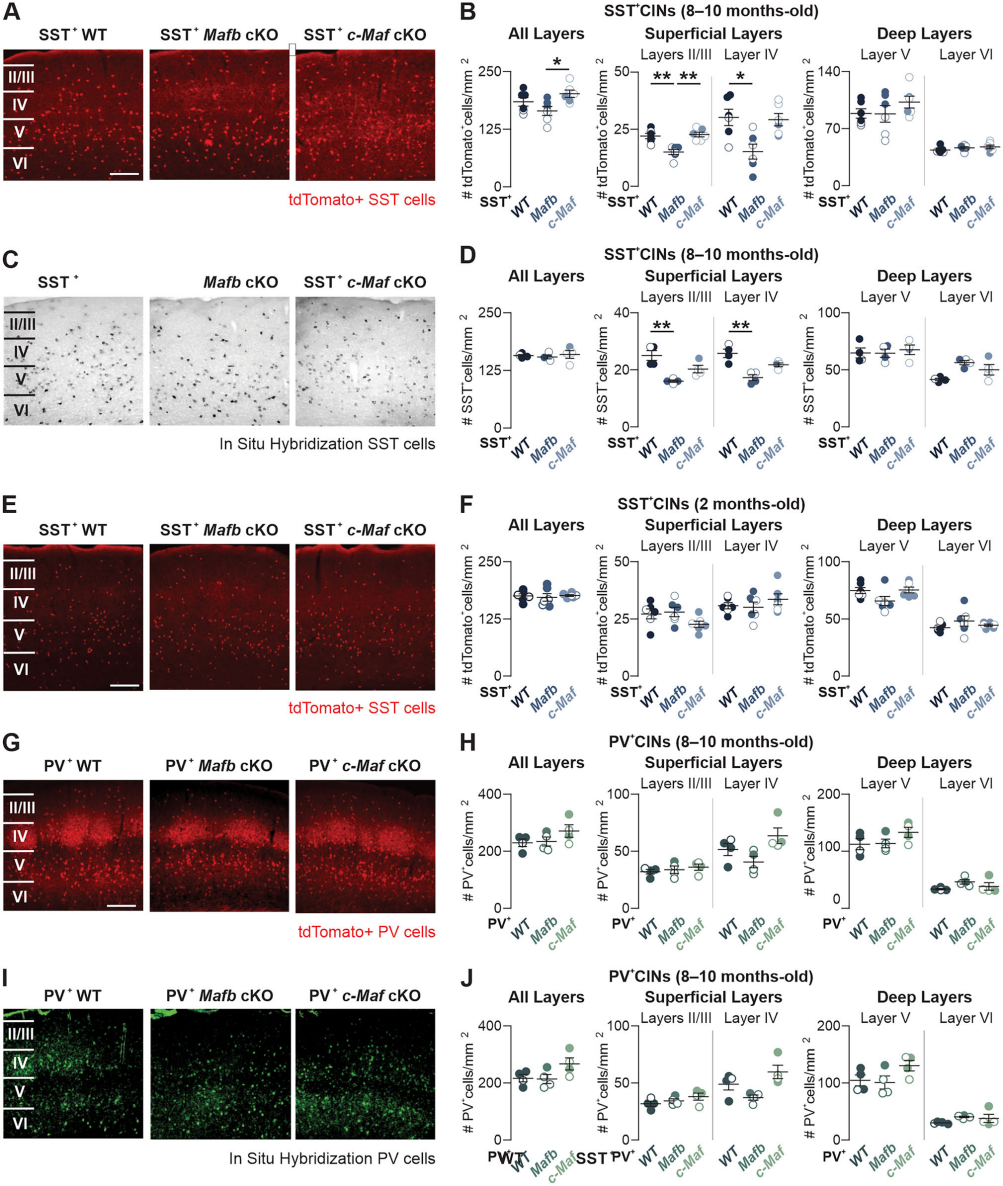
Deletion of Mafb or c-Maf differentially affects SST^+^ but not PV^+^ CIN density in the S1 cortex. ***A***, Immunofluorescent images from WT, SST^+^ *Mafb* cKO, and SST^+^ *c-Maf* cKO mice showing SST^+^ CINs marked with tdTomato (8–10-month-old mice). ***B***, Density of tdTomato^+^ cells. These results were obtained from mice age-matched with those subjected to in vivo ECoG recordings in Figure 1. *n* = 6 mice per genotype. WT, *n* = 4 males, 2 females; SST^+^ *Mafb* cKO, *n* = 3 males, 3 females; SST^+^ *c-Maf* cKO, *n* = 2 males, 4 females. ***C***, SST ISH from WT, *Mafb* cKO, and *c-Maf* cKO mice. ***D***, Density of SST^+^ CINs (8–10-month-old mice, *n* = 4 per genotype). WT, *n* = 3 males, 1 female; SST^+^ *Mafb* cKO, *n* = 2 males, 2 females; SST^+^ *c-Maf* cKO, *n* = 1 male, 3 females. ***E***, Immunofluorescent images from WT, SST^+^ *Mafb* cKO, and SST^+^ *c-Maf* cKO mice showing CINs marked with tdTomato (2-month-old mice). ***F***, Quantification of tdTomato^+^ cells/square millimeters. *n* = 6 mice per genotype. WT, *n* = 4 males, 2 females; SST^+^ *Mafb* cKO, *n* = 3 males, 3 females; SST^+^ *c-Maf* cKO, *n* = 4 males, 2 females. The effect of Mafb or c-Maf deletion in SST^+^ subtype numbers in the S1 cortex is detailed in Extended Data Figure 3-1. ***G***, Immunofluorescent images from WT, PV^+^ *Mafb* cKO, and PV^+^ *c-Maf* cKO mice showing PV^+^ CINs marked with tdTomato. ***H***, Quantification of tdTomato^+^ cells/square millimeters (8–10-month-old mice, *n* = 4 per genotype). WT, *n* = 3 males, 1 female; PV^+^ *Mafb* cKO, *n* = 1 male, 3 females; PV^+^ *c-Maf* cKO, *n* = 2 males, 2 females. ***I***, Immunofluorescent images from the S1 cortex showing immunolabeled PV^+^ CINs in green. ***J***, Quantification of PV^+^ CINs/square millimeters (8–10-month-old mice, *n* = 4 per genotype). WT, *n* = 3 males, 1 female; PV^+^ *Mafb* cKO, *n* = 1 male, 3 females; PV^+^ *c-Maf* cKO: *n* = 2 males, 2 females. Cell density was quantified using either genetic labeling (tdTomato) or marker-based IHC/ISH depending on genotype. The total (all-layers) comparison was assessed by one-way ANOVA followed by pairwise *t* tests with Holm–Šidák correction (*k* = 3 pairwise genotype contrasts). The per-layer comparison was assessed by linear mixed-effects regression (density ∼ Genotype + (1|mouse_id)) at each cortical layer, with Holm–Šidák correction across all 12 simple effects (3 pairwise genotype contrasts × 4 layers) within each panel (**p < 0.01; *p < 0.05; ns, not significant). Scale bars, 200 µm. Males are represented in gray dots and females in white dots. Each group contained male and female mice. See Extended Data Figure 3-1 for SST^+^ cell subtypes and Extended Data Figure 1-1 for complete statistical reporting.

To uncover why *c-Maf* deletion in SST^+^ CINs can lead to absence-like epileptiform activity, we first investigated its effect on the number of SST^+^ CINs. To assay the density of SST^+^ CINs, we used tdTomato IHC (Ai14 Cre reporter line crossed with *SST-Cre*) and *Sst* ISH in juvenile/young adult (2-month-old) and adult (8–10-month old) mice. These ages were chosen to match the one used for ECoG recordings. *c-Maf* deletion in SST^+^ CINs did not affect SST^+^ CIN density at either age (Fig. 3). Thus, SWCs observed in SST^+^ *c-Maf* cKO mice are not explained by changes in SST^+^ CIN density.

10.1523/ENEURO.0257-25.2026.f3-1 Figure 3-1 **Effect of Mafb or c-Maf deletion on SST^+^ subtype numbers in S1 cortex. A,** Immunofluorescent images from WT, SST^+^ *Mafb* cKO, and SST^+^ *c-Maf* cKO mice showing Calretinin + CINs (8–10-month-old mice). **B,** Quantification of Martinotti cells (double positive for tdTomato^+^ driven by SST-Cre and Calretinin). These results were obtained from mice that were age-matched with those subjected to in vivo ECoG recordings in Figure 1. n = 6 mice per genotype including both male and female mice (WT: n = 4 males, 2 females; SST^+^ *Mafb* cKO: n = 3 males, 3 females; SST^+^ *c-Maf* cKO: n = 2 males, 4 females). **C,** Immunofluorescent images showing immunolabeled nNOS^+^ CINs. **D,** Quantification of long-range SST^+^ projection neurons (double positive for tdTomato^+^ driven by SST-Cre and nNOS) in 8–10-month-old mice. n = 3 mice per genotype including both male and female mice. WT: n = 2 males, 1 female; SST^+^ *Mafb* cKO: n = 2 males, 1 female; SST^+^ *c-Maf* cKO: n = 1 male, 2 females. Statistical significance was assessed by one-way ANOVA followed by pairwise t-tests with Holm–Šidák correction (k = 3 pairwise genotype contrasts) at the all-layers level; parametric assumptions verified by Shapiro–Wilk and Levene tests (***p < 0.001; **p < 0.01; *p < 0.05). Males are represented by gray dots and females by white dots. Scale bars: 200 μm. See Figure 1-1 for complete statistical reporting. Download Figure 3-1, TIF file.

On the other hand, *Mafb* deletion in SST^+^ cells reduced SST^+^ CIN density in adult, but not juvenile mice (Fig. 3). This reduction occurred in layers II/III and IV but not in layers V and VI. Moreover, we found that the decrease in *Mafb* mutants corresponded to a reduction in Martinotti cells (SST^+^, calretinin^+^) rather than in long range-projecting SST^+^ neurons (SST^+^, nNOS^+^; Extended Data Fig. 3-1). Interestingly, *c-Maf* deletion in SST^+^ neurons increased the proportion of Martinotti cells among SST^+^ interneurons. Despite the reduction in SST^+^ CIN density, *Mafb* cKO mice do not exhibit epileptiform activity. tdTomato (Ai14, *PV-Cre*–driven) labeling and PV IHC were used to determine whether c-Maf or Mafb regulates PV^+^ CIN numbers. Deletion of neither *c-Maf n*or *Mafb* in PV^+^ cells altered PV^+^ CIN density in the adult S1 cortex (Fig. 3). Therefore, *c-Maf* and *Mafb* do not regulate PV^+^ CIN numbers in adults.

### *c-Maf* or *Mafb* deletion in SST^+^ or PV^+^ interneurons has minimal effects on active and passive membrane properties

We next investigated whether *c-Maf* deletion alters the electrophysiological properties of SST^+^ CINs. The measurements were performed in younger animals to isolate cell-intrinsic and synaptic mechanisms, independent of potential secondary network adaptations that emerge in older animals.

**Figure 4.**
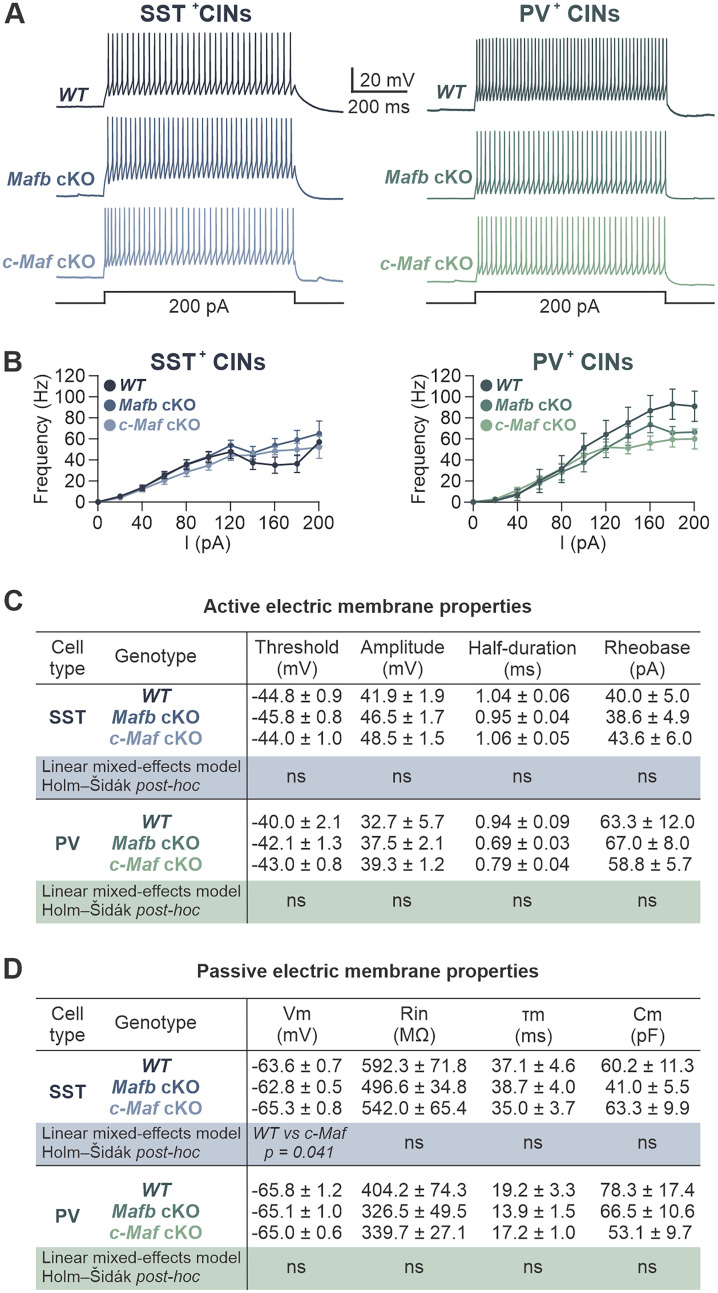
*Mafb* or *c-Maf* deletion does not affect the intrinsic membrane properties of SST^+^ and PV^+^ CINs compared with wild-type littermates. ***A***, Representative firing traces in response to a positive current injection in CINs for each neuronal type and genotype. ***B***, F–I curves showing action potential firing frequency as a function of injected current in CINs of WT, SST^+^ *Mafb* cKO, SST^+^ *c-Maf* cKO, PV^+^ *Mafb* cKO, and PV^+^ *c-Maf* cKO mice. F–I curves did not differ significantly across genotypes for either cell type at any current step or in the omnibus interaction model (all ns). ***C, D***, Quantification of active (***C***) and passive (***D***) intrinsic membrane properties. Vm, resting membrane potential; Rin, input resistance; *τ*m, membrane time constant; Cm, capacitance. Statistical significance was assessed by linear mixed-effects regression with mouse as a random intercept. For the F–I curves (***B***), spike count was modeled at each current step, and the three pairwise genotype contrasts were corrected by Holm–Šidák across the nine current steps within each cell type (*k* = 27; 3 contrasts × 9 steps). For the active (***C***) and passive (***D***) membrane properties, each measurement was modeled separately, and the three pairwise genotype contrasts were corrected by Holm–Šidák within each measurement (*k* = 3; *p < 0.05; ns, not significant). See Materials and Methods for details. All electrophysiological properties were measured in 3–5-month-old mice. In total, recordings were obtained from 134 CINs across 19 mice. SST^+^ WT, *n* = 26 cells from three mice; SST^+^ *Mafb* cKO, *n* = 29 cells from three mice; SST^+^ *c-Maf* cKO, *n* = 22 cells from two mice (both female); PV^+^ WT, *n* = 6 cells from three mice; PV^+^ *Mafb* cKO, *n* = 17 cells from four mice; PV^+^ *c-Maf* cKO, *n* = 34 cells from four mice. All measures reach *n* = 134 except Cm, where three SST^+^ WT cells lacked a usable capacitance test. See Extended Data Figure 1-1 for complete statistical reporting.

We first measured the mean firing frequency in SST^+^ CINs in response to intracellular current injections of increasing intensity in 2-month-old mice (Fig. 4*A*,*B*). Removing *c-Maf* from SST^+^ CINs did not alter their firing frequency (Fig. 4*A*) nor did it affect action potential threshold, half-duration, and rheobase (Fig. 4*C*). Removing *Mafb* from SST^+^ CINs did not have detectable effects on the firing properties of these cells (Fig. 4*A*–*C*). Among the passive electrical membrane properties in the same cells in response to intracellular negative current injections, input resistance, membrane time constant, and capacitance were not significantly altered (Fig. 4*D*). Resting membrane potential was slightly hyperpolarized in SST^+^ *c-Maf* cKO compared with SST^+^ *Mafb* cKO but not compared with WT littermates.

Removing *c-Maf* or *Mafb* in PV^+^ CINs did not affect their firing frequencies across current intensities (Fig. 4*B*). Furthermore, the action potential threshold, amplitude, and rheobase (Fig. 4*C*), as well as passive electrical membrane properties, were similar in the three genotypes (Fig. 4*D*), suggesting that *c-Maf* or *Mafb* deletions do not affect the passive membrane properties in these cells.

### *c-Maf* deletion is associated with reduced excitatory synaptic input onto SST^+^ but not PV^+^ CINs

**Figure 5.**
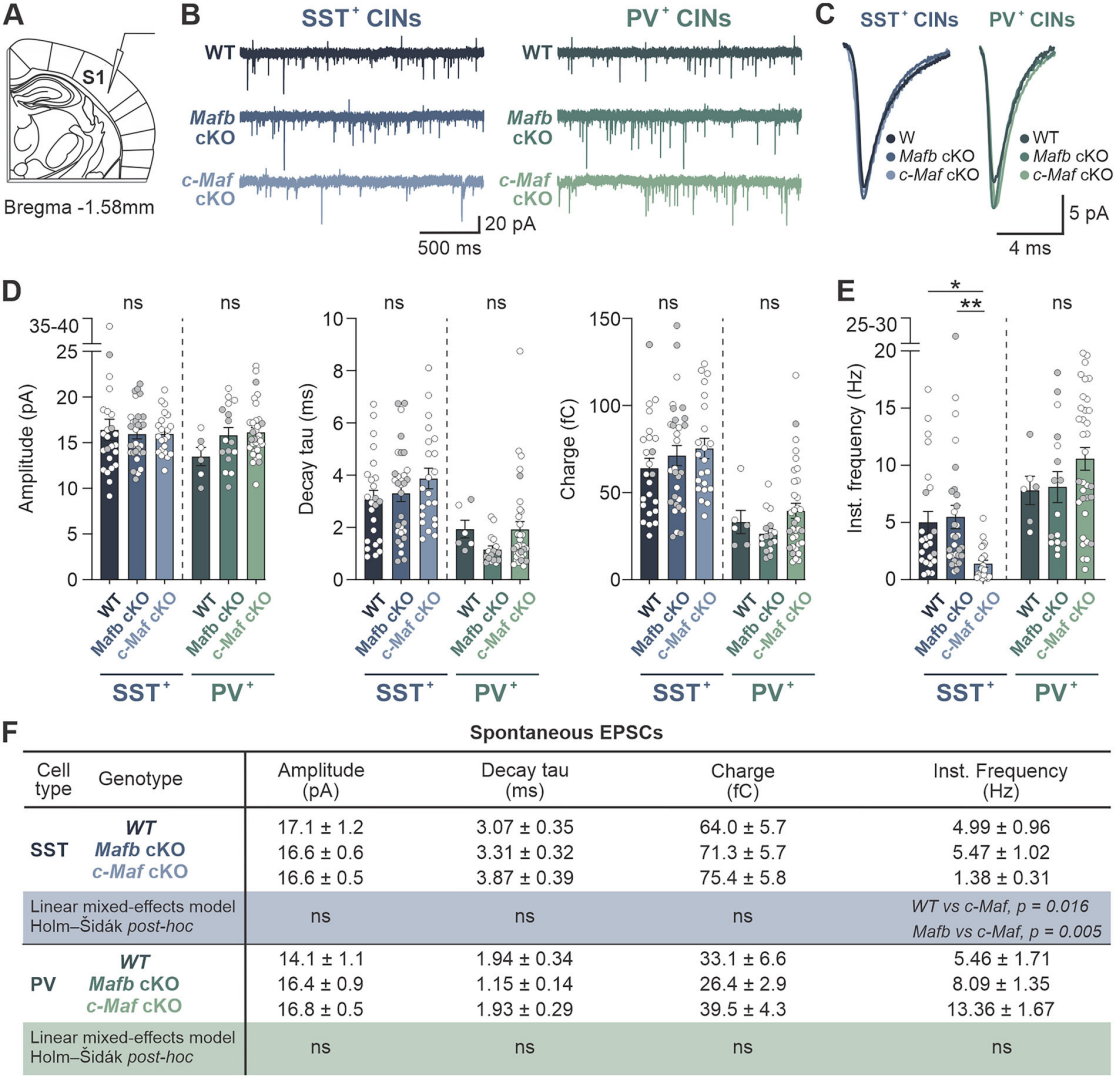
Deletion of c-Maf, but not Mafb, is associated with reduced sEPSCs in SST^+^ CINs but does not affect PV^+^ CINs. ***A***, Acute brain slice of the S1 cortex prepared for whole-cell recordings of CINs from WT, SST^+^ *Mafb* cKO, SST^+^ *c-Maf* cKO, PV^+^ *Mafb* cKO, and PV^+^ *c-Maf* cKO mice. ***B***, Representative traces of sEPSCs from CINs in S1 layer II/III. ***C***, Overlaid average sEPSCs from the representative cells depicted in ***B***. ***D***, ***E***, Quantification (mean ± SEM) of the amplitude, decay time constant, charge (***D***), and instantaneous frequency (***E***) of sEPSCs (*V*_hold_ = −70 mV) in CINs. ***F***, Summary quantification of sEPSC properties across genotypes (mean ± SEM). Statistical significance was assessed by linear mixed-effects regression with mouse as a random intercept, with Holm–Šidák correction across the three pairwise genotype contrasts (**p < 0.01; *p < 0.05; ns, not significant). See Materials and Methods for details. All electrophysiological properties were measured in 3–5-month-old mice. Males are represented by gray dots and females by white dots (note, the SST^+^ *c-Maf* cKO cohort includes only female mice). SST^+^ WT, *n* = 26 cells from three mice; SST^+^ *Mafb* cKO, *n* = 29 cells from three mice; SST^+^ *c-Maf* cKO, *n* = 22 cells from two mice. PV^+^ WT, *n* = 6 cells from three mice; PV^+^ *Mafb* cKO, *n* = 17 cells from four mice; PV^+^ *c-Maf* cKO, *n* = 34 cells from four mice. See Extended Data Figure 5-1 for related synapse count analysis and Extended Data Figure 1-1 for complete statistical reporting.

We then examined whether excitatory inputs onto CINs were affected by *c-Maf* deletion by measuring sEPSCs (Fig. 5*A*,*B*). The amplitude, decay time constant, and charge of sEPSCs in SST^+^ CINs were similar between SST^+^ *Mafb* cKO, SST^+^ *c-Maf* cKO, and SST^+^ wild-type mice (Fig. 5*C*,*D*). However, the instantaneous frequency of sEPSC in SST^+^ CINs was reduced in SST^+^ *c-Maf* cKO compared with SST^+^ wild-type and SST^+^ *Mafb* cKO CINs (Fig. 5*E*). These observations suggest that removal of *c-Maf* in SST^+^ CINs reduces the frequency of excitatory synaptic input onto SST^+^ CINs, whereas removal of *Mafb* has relatively little effect.

10.1523/ENEURO.0257-25.2026.f5-1 Figure 5-1 **Exploratory analysis of the effect of Mafb or c-Maf deletion in SST^+^ CINs on the density of excitatory synapses on their proximal dendrites in adult mice. A,** Immunofluorescent images from the S1 cortex of WT, SST^+^ *Mafb* cKO, and SST^+^ *c-Maf* cKO mice. CINs are marked with tdTomato and excitatory synapses are marked with vGlut1 and PSD95. **B,** Synapse density (puncta per pixel) on tdTomato + dendrites of SST + CINs in S1 cortex, shown as overlaid violin (kernel density), box (median and IQR), and individual dendrite measurements. Open symbols are individual dendrites color-coded by mouse of origin (circle = mouse 1, square = mouse 2); filled symbols are the mean of each mouse's dendrites; horizontal line is the average of the two mouse means per genotype. Lower sub-panel (Δ vs WT): mean difference relative to WT estimated by linear mixed-effects regression, with error bars showing ± 1 SD of the per-mouse means within each cKO group; dashed gray line marks the WT reference (Δ = 0). **C,** Empirical cumulative distribution functions (circles) and maximum-likelihood lognormal fits (solid lines) of dendritic synaptic puncta density per genotype. The *c-Maf* cKO distribution shows a ∼48% reduction in median puncta density relative to WT (0.143 vs 0.275 puncta/pixel). Statistical significance was assessed by pairwise likelihood-ratio tests on lognormal fits at the dendrite level (Bonferroni-corrected) and confirmed by linear mixed-effects regression and one-way ANOVA on per-mouse means; only the dendrite-level test reached significance for *c-Maf* cKO vs WT (*p = 0.013); all other contrasts were ns (*p < 0.05; ns, not significant). See Methods for details. Synaptic puncta analyses were performed in two-month-old mice, consistent with the age used for patch-clamp electrophysiology experiments in Figures 4 and 5. n = 10 dendrites from 2 mice (one male, one female) per genotype (30 dendrites from 6 mice total). Scale bar: 5 μm. See Figure 1-1 for complete statistical reporting. Download Figure 5-1, TIF file.

To assess whether the reduction in sEPSC frequency in SST^+^ *c-Maf* cKO neurons might reflect, at least in part, changes in excitatory synapse number, we quantified excitatory synapses on SST^+^ CINs using vGlut1 and PSD95 markers (Extended Data Fig. 5-1*A*). This anatomical analysis was performed on a limited number of biological replicates (*n* = 2 mice per genotype) and should therefore be interpreted as exploratory rather than fully powered. At the level of sampled dendrites (*n* = 10 per genotype, sampled across two biological replicates), the distribution of excitatory synapse density was visibly shifted toward lower values in SST^+^ *c-Maf* cKO mice compared with both WT and SST^+^ *Mafb* cKO controls (Extended Data Fig. 5-1*B*), with *c-Maf* cKO dendrites populating the left tail of the cumulative distribution and reaching the upper plateau at lower puncta densities. The dendritic distributions were well described by lognormal fits, and a likelihood-ratio test indicated that the *c-Maf* cKO distribution differed significantly from WT at the dendrite level, corresponding to an ∼48% reduction in median dendritic puncta density; this difference did not reach significance when the mouse was treated as the unit of analysis (Extended Data Fig. 5-1*B*), and we therefore interpret it as exploratory. The *Mafb* cKO distribution was not significantly different from either WT or *c-Maf* cKO, consistent with an intermediate phenotype. While changes in sEPSC frequency can also reflect presynaptic release probability, these structural data are consistent with, but do not independently establish, reduced excitatory synapse density. Together with the electrophysiological findings, these data suggest that reduced excitatory synapse numbers may contribute to the decreased synaptic excitation in SST^+^ *c-Maf* cKO CINs.

In PV^+^ CINs, removal of *Mafb* or *c-Maf* had no effect on the amplitude, decay time constant, charge (Fig. 5*C*,*D*), or instantaneous frequency of sEPSCs (Fig. 5*E*,*F*). These results indicate that *Mafb* or *c-Maf* deletions in PV^+^ CINs do not alter their excitatory synaptic input.

## Discussion

CINs play an important role in controlling local circuit functions in part through regulating the excitation/inhibition balance, and their disruption leads to epilepsy and other disorders (Rubenstein and Merzenich, 2003; Chao et al., 2010; Han et al., 2012). Whether specific subtypes of CINs contribute to these disorders is not understood. Previous work showed that disrupting development of MGE-derived PV^+^ and SST^+^ CINs by deleting the Mafb and c-Maf TFs before the specification of their SST^+^ versus PV^+^ fate resulted in electrographic seizures (Pai et al., 2020), but the relative contributions of Mafb and c-Maf in SST^+^ versus PV^+^ cells to these seizures remained unknown. Here, we individually deleted these TFs in either PV^+^ or SST^+^ cells to determine their cell type-specific roles in epileptiform discharges. We found that deletion of c-Maf in SST^+^ cells led to absence-like SWDs in female and male mice, whereas deletion of Mafb in SST^+^ cells or deletion of either c-Maf or Mafb in PV^+^ cells did not. Furthermore, we found important differences in the functions of c-Maf and Mafb in regulating the biology of SST^+^ CINs.

The finding that deleting *c-Maf* in SST^+^ (Fig. 1), but not in PV^+^ cells (Fig. 2), results in absence-like epileptiform activity is intriguing because PV^+^ CINs are thought to provide stronger perisomatic inhibition of pyramidal neurons than SST^+^ CINs (Pfeffer et al., 2013) and are often implicated in epilepsy (Sessolo et al., 2015; Jiang et al., 2016; Godoy et al., 2022). SST^+^ interneurons, although less dominant in perisomatic inhibition, nonetheless play important roles in shaping feedforward inhibition and restraining seizure propagation and ictal dynamics in cortical and hippocampal networks (Parrish et al., 2019; Wengert et al., 2021; Drexel et al., 2022), suggesting that disruption of SST^+^ function alone can destabilize network activity.

c-Maf deletion in SST^+^ cells did not alter cell number or most intrinsic membrane properties (Figs. 3, 4), although SST^+^ CINs exhibited a hyperpolarized resting membrane potential compared with Mafb SST cKO CINs. These findings suggest that the epileptiform phenotype of SST^+^ *c-Maf* cKO mice is associated with altered excitatory synaptic drive rather than with broad changes in interneuron abundance or intrinsic membrane properties. Reduced excitatory synaptic input, together with a slightly hyperpolarized membrane potential, would both be expected to decrease SST^+^ CIN recruitment during network activity, thereby reducing their overall excitability. This interpretation is consistent with prior studies showing that diminished SST^+^ interneuron engagement can facilitate network hyperexcitability and seizure propagation (Parrish et al., 2019; Wengert et al., 2021; Drexel et al., 2022). The mechanism by which c-Maf influences excitatory synaptic input but may involve transcriptional control of synaptogenic pathways, including heparan sulfate proteoglycans such as Hs3st1, which is expressed in SST^+^ CINs and implicated in synapse formation (Paul et al., 2017; Condomitti and de Wit, 2018). Notably, c-Maf, but not Mafb, regulates Hs3st1 expression in other neuronal systems (Bastille et al., 2025), raising the possibility that a similar pathway contributes here.

The slight and unexplained increase in PV^+^ CINs observed in SST^+^ *c-Maf* cKO mice (Fig. 3) was insufficient to prevent epileptiform activity, suggesting that changes in SST^+^ CIN function are associated with SWDs in this model despite a modest increase in PV^+^ interneuron density.

The absence of epileptiform activity following c-Maf deletion in PV^+^ cells may reflect differences in recombination timing and/or developmental roles, intrinsic transcriptional roles across interneuron subtypes, or compensatory mechanisms specific to PV^+^ networks. Together, these findings suggest a greater sensitivity of SST^+^ CINs to c-Maf loss after fate specification.

Because the nucleus reticularis thalami (nRT) plays a central role in generating SWDs in absence epilepsy (Crunelli and Leresche, 2002; Slaght et al., 2002; Blumenfeld, 2005; Paz and Huguenard, 2015; Fogerson and Huguenard, 2016), its potential contribution to the phenotype reported here warrants consideration. The nRT is composed of PV^+^ and SST^+^ neurons (Clemente-Perez et al., 2017; Vantomme et al., 2019; Martinez-Garcia et al., 2020; Hoseini et al., 2021). Because *Somatostatin-IRES-Cre* is not restricted to the cortex, c-Maf would also have been deleted in these SST^+^ nRT neurons, so a thalamic contribution cannot be formally excluded. Three considerations, however, argue against a primary nRT-driven mechanism in this model. First, from an ontogenetic perspective, Maf TFs (*c-Maf* and *Mafb*) are established regulators of telencephalic interneuron development (Pai et al., 2019, 2020) and are not recognized as primary drivers of diencephalic nRT specification. Second, the nRT is mainly composed of PV^+^ neurons (Clemente-Perez et al., 2017; Vantomme et al., 2019; Martinez-Garcia et al., 2020), but our PV^+^ c-Maf and Mafb cKO mice did not develop SWDs (Fig. 2). Third, prior functional dissection of nRT subtypes showed that PV^+^, but not SST^+^, nRT neurons are rhythmogenic and able to modulate seizures, whereas SST^+^ nRT neurons instead gate sensory and attentional thalamocortical signaling (Clemente-Perez et al., 2017; Hoseini et al., 2021). These considerations favor a primarily telencephalic rather than diencephalic SST^+^ mechanism, although a modulatory contribution from nRT neurons remains possible and merits future investigation.

More broadly, our overall results suggest that *Mafb* and *c-Maf* regulate different aspects of the biology of SST^+^ cells. Indeed, loss of *c-Maf* function in SST^+^ CINs reduced sEPSC frequency (Fig. 5), consistent with a tendency to reduce excitatory synapse numbers (Extended Data Fig. 5-1), whereas loss of *Mafb* function in these interneurons did not result in these phenotypes (Fig. 5, Extended Data Fig. 5-1). Similarly, removing *c-Maf* function in MGE progenitors was associated with increased neurite complexity, but loss of *Mafb* function at this stage did not affect neurite complexity (Pai et al., 2020). On the other hand, deletion of *Mafb* led to a reduction in SST^+^ CIN density in 8–10-month-old mice (Fig. 3), but deletion of *c-Maf* did not. Thus, loss of *Mafb* results in an age-dependent loss of CIN numbers, similar to that observed in *Dlx1* mutants (Cobos et al., 2005), and in certain CIN-selective neurodegenerative disorders.

In addition, Mafb and c-Maf appear to differentially regulate SST^+^ interneuron subtypes (Extended Data Fig. 3-1). Mafb deletion reduced the number of Martinotti cells, whereas c-Maf deletion altered the relative proportion of SST^+^ subtypes, including Martinotti cells. These findings suggest that Maf family TFs contribute to the diversification and maintenance of SST^+^ interneuron subtypes after fate specification, which may underlie differences in circuit impact and contribute to functional heterogeneity within SST^+^ populations.

One structural difference between Mafb and c-Maf is the presence of a GSK3 phosphorylation motif in the transactivation domain of c-Maf, which is absent in Mafb (Niceta et al., 2015). This motif regulates c-Maf activity and has been implicated in human neurodevelopmental disease, as mutations that disrupt GSK3-mediated phosphorylation of c-MAF cause Aymé–Gripp syndrome, which includes intellectual disability and seizures (Niceta et al., 2015). These findings suggest that differential posttranslational regulation of c-Maf may contribute to its unique roles in SST^+^ interneurons, although this remains to be directly tested.

### Limitations

This study integrates analyses performed at different developmental stages, including patch-clamp recordings in younger animals (2–5 months) and in vivo ECoG in slightly older mice (8–10 months), with all comparisons restricted to within-age, genotype-matched cohorts; however, this limits direct quantitative comparisons across modalities and precludes inference about developmental trajectories. Differences in Cre recombination timing between SST^+^ and PV^+^ lines, as well as limited statistical power to assess sex as a biological variable, may also contribute to phenotype variability across conditions. In addition, synaptic analyses were performed on a limited number of biological replicates and should be interpreted as supportive. Finally, while our study focuses on SST^+^ interneurons, contributions from other cell types or brain regions cannot be excluded. Although our data are consistent with a model in which reduced excitatory drive onto SST^+^ interneurons contributes to the spike-and-wave phenotype, we did not directly measure SST^+^ inhibitory output, and a causal link between these synaptic changes and SWDs remains to be established. Future studies will be required to determine whether the observed synaptic and network alterations are restricted to SST^+^ CINs or reflect broader circuit-level changes.

In spite of these limitations, our results indicate that SST^+^ interneurons are sensitive to perturbations of c-Maf during development, an effect that correlates with the emergence of absence-like epileptiform activity. Future work is warranted to define the molecular, cellular, and circuit mechanisms underlying this phenotype, as well as the basis of the distinct effects of Mafb and c-Maf on SST^+^ interneuron populations. Overall, these results identify SST^+^ CIN dysfunction as a potential contributor to epileptiform network dysfunction and provide a framework for exploring c-MAF–dependent mechanisms in disease.

## Synthesis

Reviewing Editor: Catherine Schevon, Columbia University Decisions are customarily a result of the Reviewing Editor and the peer reviewers coming together and discussing their recommendations until a consensus is reached. When revisions are invited, a fact-based synthesis statement explaining their decision and outlining what is needed to prepare a revision will be listed below. The following reviewer(s) agreed to reveal their identity: R. Ryley Parrish.

The authors have made a thoughtful revision of their previous submission. The revised manuscript is improved in several important ways. In particular, the authors strengthened the statistical analysis, added methodological detail, clarified several limitations, moved the underpowered synaptic puncta analysis to Extended Data, and incorporated additional discussion of SST interneurons in seizure-related circuit function.

Main concerns

1. The sex-reporting language needs clarification. The rebuttal states that the SST+ c-Maf cKO group consisted only of female mice, but some figure legends appear to report both males and females in SST+ c-Maf cKO histology cohorts. The authors likely mean that the SST+ c-Maf cKO electrophysiology cohort lacked males, but this should be stated explicitly. As written, it remains somewhat confusing still.

2. The synaptic puncta analysis should remain clearly framed as exploratory. The authors appropriately moved the dendritic puncta analysis to Extended Data and acknowledge that the mouse-level analyses are underpowered. However, because the significant result appears to be dendrite-level rather than biological-replicate-level, the manuscript should avoid firm conclusions that c-Maf deletion reduces excitatory synapse number. A safer interpretation would be that the structural data are consistent with, but do not independently establish, reduced excitatory synapse density.

3. Some causal language should be softened. The manuscript still occasionally reads as though reduced excitatory input onto SST+ interneurons directly causes the spike-and-wave phenotype. The data support an association and a plausible model, but they do not directly test inhibitory output or establish mechanism. Please replace stronger causal phrasing with wording such as "is associated with" or "may contribute to."

4. Please add a statement to the discussion acknowledging the potential role for the thalamic reticular nucleus in the absence-like events observed in the model. Because most thalamic reticular neurons are PV positive and conditional deletion of c-Maf and Mafb in PV cells did not produce spike wave discharges it is unlikely that the thalamic reticular nucleus is causal in the generation of spike wave discharges. However, your lab's prior work has shown that a subset of neurons within the thalamic reticular nucleus are SST positive and PV negative which raises the possibility that this subset of neurons is contributing to the spike wave discharges observed in the SST-specific conditional KO c-MAF group. Given that and the established role of the thalamus in absence epilepsy, this possibility should be noted.

Minor comments

1. Clean up repeated or awkward figure legend text, including the duplicated wording about overlaid fast Fourier transforms in the Figure 1 legend.

2. Standardize terminology across the manuscript, where the authors use SWDs, SWCs, spike-and-wave discharges, epileptiform activity, and absence-like seizures. Or, clearly clarify the differences.
