# Supplementary material for: c-Maf Deletion in Cortical Somatostatin, But Not Parvalbumin, Interneurons Leads to Absence-Like Epileptiform Activity in Mice
Source: eNeuro. 2026 Jul 17;13(7):ENEURO.0257-25.2026. doi: 10.1523/ENEURO.0257-25.2026 (PMC13395531; doi:10.1523/ENEURO.0257-25.2026)
Supplement: Figure 1-1 — Statistical Summary Table. This table provides a comprehensive overview of the statistical analyses and descriptive metrics corresponding to the experimental data presented in Figures 1 through 5 (including extended data panels). Data are derived from wildtype (WT), Mafb conditional knockout (Mafb cKO), and c-Maf conditional knockout (c-Maf cKO) mouse lines within both Somatostatin-cre and Parvalbumin-cre cohorts of mice. Descriptive statistics are presented as mean ± SEM unless otherwise noted. Abbreviations and definitions: Genotypes and Cohorts: WT: Wildtype control; Mafb cKO: Mafb conditional knockout; c-Maf cKO: c-Maf conditional knockout; SST+: Somatostatin-expressing; PV+: Parvalbumin-expressing; CIN: Cortical interneuron. Anatomical and histological terms: PFC: Prefrontal cortex; S1: Primary somatosensory cortex; Layers II/III, IV, V, VI: Specific neocortical layers; ISH: In situ hybridization; tdTomato: Fluorescent reporter protein used for genetic cell lineage tracking; nNOS+: Neuronal nitric oxide synthase-expressing (marking long-range SST + neurons). Electrophysiological parameters: AP: Action potential; Vm: Resting membrane potential (measured in mV); Rin: Input resistance (measured in MΩ); τm (tau_m): Membrane time constant (measured in ms); Cm: Membrane capacitance (measured in pF); Rheobase: Minimum current required to elicit an action potential (measured in pA); sEPSC: Spontaneous excitatory postsynaptic current; Decay tau: Synaptic current decay time constant. Units of Measurement: mo: months of age; Hz: Hertz (cycles per second); pA: picoamperes; fC: femtocoulombs; ms / mV: milliseconds / millivolts. Statistical nomenclature and significance thresholds: n: sample size (explicitly indicated as number of mice, sections, or individual patched cells where appropriate); ANOVA: Analysis of variance (used for parametric group comparisons); Kruskal–Wallis (K–W): Non-parametric equivalent to one-way ANOVA used for non-normally distributed data; LMM: Linea [file eneuro-13-ENEURO.0257-25.2026-s005.docx]

**Figure 1-1. Statistical Summary Table related to Figures 1-5.**

| **Figure** | **Description** | **Test** | **n (per group)** | **Statistic** | **p-value** | **Mean ± SEM** |
| --- | --- | --- | --- | --- | --- | --- |
| 1B | Epileptiform spikes/hour, PFC, SST+ cohort, 8–9 mo | Kruskal–Wallis + Dunn's multiple-comparisons (Bonferroni-adjusted) | WT: 7 mice  Mafb cKO: 10 mice  c-Maf cKO: 6 mice | Kruskal–Wallis: H(2) = 12.90  Dunn's z:  WT vs Mafb cKO: z = −0.48  WT vs c-Maf cKO: z = −3.29  Mafb cKO vs c-Maf cKO: z = −3.09 | **K–W: p = 0.0016 ****  Dunn's (Bonferroni):  WT vs Mafb cKO: p > 0.99 (n.s.)  **WT vs c-Maf cKO: p = 0.003 ****  **Mafb cKO vs c-Maf cKO: p = 0.006 **** | WT: 0.29 ± 0.19  Mafb cKO: 0.77 ± 0.39  c-Maf cKO: 16.06 ± 10.77 |
| 1C | Epileptiform spikes/hour, S1, SST+ cohort, 8–9 mo | Kruskal–Wallis + Dunn's multiple-comparisons (Bonferroni-adjusted) | WT: 7 mice  Mafb cKO: 10 mice  c-Maf cKO: 6 mice | Kruskal–Wallis: H(2) = 9.61  Dunn's z:  WT vs Mafb cKO: z = −1.62  WT vs c-Maf cKO: z = −3.10  Mafb cKO vs c-Maf cKO: z = −1.80 | **K–W: p = 0.008 ****  Dunn's (Bonferroni):  WT vs Mafb cKO: p = 0.32 (n.s.)  **WT vs c-Maf cKO: p = 0.006 ****  Mafb cKO vs c-Maf cKO: p = 0.22 (n.s.) | WT: 1.47 ± 1.47  Mafb cKO: 2.06 ± 0.58  c-Maf cKO: 24.19 ± 17.40 |
| 2A | Epileptiform spikes/hour, PFC, PV+ cohort, 8–9 mo | Kruskal–Wallis + Dunn's multiple-comparisons (Bonferroni-adjusted) | WT: 5 mice  Mafb cKO: 9 mice  c-Maf cKO: 5 mice | Kruskal–Wallis: H(2) = 2.75  Dunn's z:  WT vs Mafb cKO: z = −0.84  WT vs c-Maf cKO: z = −1.65  Mafb cKO vs c-Maf cKO: z = −1.03 | K–W: p = 0.25 (n.s.)  Dunn's (Bonferroni):  WT vs Mafb cKO: p > 0.99 (n.s.)  WT vs c-Maf cKO: p = 0.29 (n.s.)  Mafb cKO vs c-Maf cKO: p = 0.91 (n.s.) | WT: 0.00 ± 0.00  Mafb cKO: 0.17 ± 0.11  c-Maf cKO: 0.49 ± 0.31 |
| 2B | Epileptiform spikes/hour, S1, PV+ cohort, 8–9 mo | Kruskal–Wallis + Dunn's multiple-comparisons (Bonferroni-adjusted) | WT: 5 mice  Mafb cKO: 9 mice  c-Maf cKO: 6 mice | Kruskal–Wallis: H(2) = 1.81  Dunn's z:  WT vs Mafb cKO: z = 0.82  WT vs c-Maf cKO: z = −0.37  Mafb cKO vs c-Maf cKO: z = −1.30 | K–W: p = 0.40 (n.s.)  Dunn's (Bonferroni):  WT vs Mafb cKO: p > 0.99 (n.s.)  WT vs c-Maf cKO: p > 0.99 (n.s.)  Mafb cKO vs c-Maf cKO: p = 0.58 (n.s.) | WT: 0.39 ± 0.24  Mafb cKO: 0.21 ± 0.14  c-Maf cKO: 0.89 ± 0.49 |
| 3B | SST+ CIN density, S1, adult (tdTomato), per layer | All layers: One-way ANOVA + pairwise t-tests, Holm–Šidák k = 3 (parametric: Shapiro p > 0.05, Levene p = 0.63)  Per-layer LMM (density ~ Genotype + (1\|mouse_id)) at each cortical layer; Holm–Šidák k = 12 across (3 pairwise contrasts × 4 layers) within panel. | 6 mice/genotype; 2–3 sections/mouse | All layers:  ANOVA: F(2, 15) = 4.23  Mafb − WT: t(10) = −1.46  c-Maf − WT: t(10) = +1.42  c-Maf − Mafb: t(10) = +2.96  Per-layer:  (Mafb−WT / c-Maf−WT / c-Maf−Mafb):  Layer II/III: t(15) = −4.45 / +0.42 / +4.87  Layer IV: t(15) = −3.31 / −0.22 / +3.09  Layer V: t(15) = −0.04 / +1.24 / +1.28  Layer VI: t(15) = +1.07 / +1.54 / +0.47 | All layers:  **ANOVA: p = 0.035 ***  Mafb − WT: p = 0.320  c-Maf − WT: p = 0.320  **c-Maf − Mafb: p = 0.042 ***  Per layer:  Holm–Šidák k = 12:  **Layer II/III: p = 0.005 ** / 0.984 / 0.002 ****  **Layer IV: p = 0.047 * / 0.984 / 0.066**  Layer V: p = 0.984 / 0.822 / 0.822  Layer VI: p = 0.832 / 0.712 / 0.984 | All layers:  WT: 184.3 ± 9.4  Mafb cKO: 164.5 ± 9.8  c-Maf cKO: 201.8 ± 7.9  Per-layer:  Layer II/III: WT 22.0 ± 1.2; Mafb 15.0 ± 1.1; c-Maf 22.7 ± 1.0  Layer IV: WT 30.2 ± 3.6; Mafb 15.2 ± 3.3; c-Maf 29.2 ± 2.7  Layer V: WT 88.5 ± 5.8; Mafb 88.0 ± 10.2; c-Maf 102.5 ± 7.3  Layer VI: WT 43.7 ± 1.5; Mafb 46.3 ± 1.4; c-Maf 47.5 ± 2.2 |
| 3D | Sst mRNA ISH cell density, adult, per layer | All layers: One-way ANOVA + pairwise t-tests, Holm–Šidák k = 3 (parametric: Shapiro p > 0.05, Levene p = 0.46)  Per-layer LMM (density ~ Genotype + (1\|mouse_id)) at each cortical layer; Holm–Šidák k = 12 across (3 pairwise contrasts × 4 layers) within panel. | 4 mice/genotype; ≥10 sections/mouse | All layers:  ANOVA: F(2, 9) = 0.25  Mafb − WT: t(6) = −0.65  c-Maf − WT: t(6) = +0.29  c-Maf − Mafb: t(6) = +0.59  Per-layer:  (Mafb−WT / c-Maf−WT / c-Maf−Mafb):  Layer II/III: t(9) = −4.98 / −2.63 / +2.35  Layer IV: t(9) = −5.42 / −2.55 / +2.87  Layer V: t(9) = −0.04 / +0.48 / +0.52  Layer VI: t(9) = +3.56 / +2.05 / −1.51 | All layers:  ANOVA: p = 0.787  Mafb − WT: p = 0.903  c-Maf − WT: p = 0.903  c-Maf − Mafb: p = 0.903  Per layer:  Holm–Šidák k = 12:  **Layer II/III: p = 0.008 ** / 0.199 / 0.232**  **Layer IV: p = 0.005 ** / 0.199 / 0.154**  Layer V: p = 0.966 / 0.943 / 0.943  Layer VI: p = 0.060 / 0.307 / 0.517 | All layers:  WT: 157.0 ± 2.4  Mafb cKO: 154.0 ± 4.0  c-Maf cKO: 159.5 ± 8.4  Per-layer:  Layer II/III: WT 25.0 ± 1.7; Mafb 16.0 ± 0.4; c-Maf 20.2 ± 1.3  Layer IV: WT 25.8 ± 1.5; Mafb 17.2 ± 1.0; c-Maf 21.8 ± 0.6  Layer V: WT 64.8 ± 4.4; Mafb 64.5 ± 3.6; c-Maf 67.5 ± 4.2  Layer VI: WT 41.5 ± 1.0; Mafb 56.2 ± 1.9; c-Maf 50.0 ± 4.6 |
| 3F | SST+ CIN density, S1, juvenile (2 mo), per layer | All layers: One-way ANOVA + pairwise t-tests, Holm–Šidák k = 3 (parametric: Shapiro p > 0.05, Levene p = 0.12)  Per-layer LMM (density ~ Genotype + (1\|mouse_id)) at each cortical layer; Holm–Šidák k = 12 across (3 pairwise contrasts × 4 layers) within panel. | 6 mice/genotype | All layers:  ANOVA: F(2, 15) = 0.15  Mafb − WT: t(10) = −0.34  c-Maf − WT: t(10) = +0.20  c-Maf − Mafb: t(10) = +0.49  Per-layer:  (Mafb−WT / c-Maf−WT / c-Maf−Mafb):  Layer II/III: t(15) = +0.32 / −1.70 / −2.02  Layer IV: t(15) = −0.23 / +0.97 / +1.20  Layer V: t(15) = −2.08 / +0.11 / +2.19  Layer VI: t(15) = +1.47 / +0.55 / −0.93 | All layers:  ANOVA: p = 0.86  Mafb − WT: p = 0.95  c-Maf − WT: p = 0.95  c-Maf − Mafb: p = 0.95  Per layer:  Holm–Šidák k = 12:  Layer II/III: p = 0.986 / 0.647 / 0.472  Layer IV: p = 0.986 / 0.923 / 0.866  Layer V: p = 0.465 / 0.986 / 0.422  Layer VI: p = 0.755 / 0.972 / 0.923 | All layers:  WT: 174.8 ± 4.6  Mafb cKO: 171.7 ± 8.2  c-Maf cKO: 175.8 ± 1.9  Per layer:  Layer II/III: WT 27.2 ± 2.1; Mafb 28.0 ± 2.0; c-Maf 22.7 ± 1.4  Layer IV: WT 30.7 ± 1.4; Mafb 30.0 ± 2.3; c-Maf 33.5 ± 2.4  Layer V: WT 74.7 ± 2.7; Mafb 65.5 ± 3.9; c-Maf 75.2 ± 2.5  Layer VI: WT 42.3 ± 1.5; Mafb 48.2 ± 4.5; c-Maf 44.5 ± 1.0 |
| 3H | PV+ density (tdTomato), S1, adult, per layer | All layers: One-way ANOVA + pairwise t-tests, Holm–Šidák k = 3 (parametric: Shapiro p > 0.05, Levene p = 0.70)  Per-layer LMM (density ~ Genotype + (1\|mouse_id)) at each cortical layer; Holm–Šidák k = 12 across (3 pairwise contrasts × 4 layers) within panel. | 4 mice/genotype | All layers:  ANOVA: F(2, 9) = 1.71  Mafb − WT: t(6) = +0.23  c-Maf − WT: t(6) = +1.62  c-Maf − Mafb: t(6) = +1.36  Per-layer:  (Mafb−WT / c-Maf−WT / c-Maf−Mafb):  Layer II/III: t(9) = +0.45 / +1.03 / +0.58  Layer IV: t(9) = −1.34 / +1.46 / +2.80  Layer V: t(9) = +0.10 / +1.68 / +1.58  Layer VI: t(9) = +2.02 / +0.75 / −1.27 | All layers:  ANOVA: p = 0.23  Mafb − WT: p = 0.83  c-Maf − WT: p = 0.40  c-Maf − Mafb: p = 0.40  Per layer:  Holm–Šidák k = 12:  Layer II/III: p = 0.924 / 0.864 / 0.924  Layer IV: p = 0.814 / 0.792 / 0.222  Layer V: p = 0.924 / 0.745 / 0.766  Layer VI: p = 0.573 / 0.922 / 0.814 | All layers:  WT: 229.3 ± 13.3  Mafb cKO: 234.0 ± 15.8  c-Maf cKO: 270.8 ± 21.9  Per-layer:  Layer II/III: WT 32.0 ± 2.0; Mafb 33.8 ± 3.3; c-Maf 36.0 ± 2.7  Layer IV: WT 51.5 ± 5.4; Mafb 40.5 ± 5.0; c-Maf 63.5 ± 6.9  Layer V: WT 112.2 ± 9.7; Mafb 113.5 ± 7.6; c-Maf 133.0 ± 8.8  Layer VI: WT 33.5 ± 1.2; Mafb 46.2 ± 3.9; c-Maf 38.2 ± 6.6 |
| 3J | PV+ density (PV immunolabel), S1, adult, per layer | All layers: One-way ANOVA + pairwise t-tests, Holm–Šidák k = 3 (parametric: Shapiro p > 0.05, Levene p = 0.66)  Per-layer LMM (density ~ Genotype + (1\|mouse_id)) at each cortical layer; Holm–Šidák k = 12 across (3 pairwise contrasts × 4 layers) within panel. | 4 mice/genotype | ANOVA: F(2, 9) = 3.12  Mafb − WT: t(6) = −0.12  c-Maf − WT: t(6) = +2.04  c-Maf − Mafb: t(6) = +2.00  Per-layer:  (Mafb−WT / c-Maf−WT / c-Maf−Mafb):  Layer II/III: t(9) = +0.72 / +1.81 / +1.08  Layer IV: t(9) = −1.78 / +1.63 / +3.41  Layer V: t(9) = −0.26 / +1.80 / +2.07  Layer VI: t(9) = +1.76 / +1.30 / −0.46 | All layers:  ANOVA: p = 0.093  Mafb − WT: p = 0.91  c-Maf − WT: p = 0.24  c-Maf − Mafb: p = 0.24  Per layer:  Holm–Šidák k = 12:  Layer II/III: p = 0.866 / 0.668 / 0.769  Layer IV: p = 0.668 / 0.668 / 0.089  Layer V: p = 0.882 / 0.668 / 0.544  Layer VI: p = 0.668 / 0.724 / 0.882 | All layers:  WT: 216.3 ± 12.6  Mafb cKO: 213.8 ± 15.8  c-Maf cKO: 266.8 ± 21.3  Per-layer:  Layer II/III: WT 31.8 ± 2.2; Mafb 34.2 ± 1.8; c-Maf 38.0 ± 3.1  Layer IV: WT 50.0 ± 5.1; Mafb 38.2 ± 2.2; c-Maf 60.8 ± 5.9  Layer V: WT 104.5 ± 9.5; Mafb 100.8 ± 11.5; c-Maf 130.2 ± 9.2  Layer VI: WT 30.0 ± 0.9; Mafb 40.5 ± 1.3; c-Maf 37.8 ± 7.1 |
| 3-1B | Martinotti cell density (SST+/Calretinin+), S1, adult | One-way ANOVA + pairwise t-tests, Holm–Šidák k = 3 (parametric: Shapiro p > 0.05, Levene p = 0.24) | 6 mice/genotype | All layers:  ANOVA: F(2, 15) = 19.94  Mafb − WT: t(10) = −3.22  c-Maf − WT: t(10) = +3.06  c-Maf − Mafb: t(10) = +6.40 | All layers:  **ANOVA: p = 6 × 10⁻⁵ ******  **Mafb − WT: p = 0.018 ***  **c-Maf − WT: p = 0.018 ***  **c-Maf − Mafb: p = 2.4 × 10⁻⁴ ***** | All layers:  WT: 27.9 ± 2.1  Mafb cKO: 17.0 ± 2.6  c-Maf cKO: 35.1 ± 1.0 |
| 3-1D | Long-range SST+ density (SST+/nNOS+), S1, adult | All layers: One-way ANOVA + pairwise t-tests, Holm–Šidák k = 3 (parametric: Shapiro p > 0.05, Levene p = 0.86) | 3 mice/genotype | All layers:  ANOVA: F(2, 6) = 0.05  Mafb − WT: t(4) = −0.23  c-Maf − WT: t(4) = +0.02  c-Maf − Mafb: t(4) = +0.30 | All layers:  ANOVA: p = 0.96  Mafb − WT: p = 0.99  c-Maf − WT: p = 0.99  c-Maf − Mafb: p = 0.99 | All layers:  WT: 3.52 ± 0.73  Mafb cKO: 3.29 ± 0.70  c-Maf cKO: 3.54 ± 0.42 |
| 4B | Action potential firing frequency vs current step, SST+ CINs | Per-step LMM (spike_count ~ Genotype + 1\|mouse_id); Holm–Šidák k = 27 across (3 contrasts × 9 current steps) within cell type. Omnibus interaction LMM additionally fit. | SST+ WT: 26 cells/3 mice  Mafb cKO: 29 cells/3 mice  c-Maf cKO: 22 cells/2 mice | Omnibus interaction:  Mafb cKO × current_pA: t(79.2) = 0.53  c-Maf cKO × current_pA: t(75.7) = −1.30  Per-step contrasts (Mafb−WT / c-Maf−WT / c-Maf−Mafb):  20 pA: t = −0.25 / −0.77 / −0.55  40 pA: t = 0.10 / −0.39 / −0.50  60 pA: t = −0.23 / −1.03 / −0.84  80 pA: t = 0.09 / −1.04 / −1.17  100 pA: t = 0.13 / −1.08 / −1.26  120 pA: t = 0.81 / −0.48 / −1.37  140 pA: t = 1.00 / 0.65 / −0.35  160 pA: t = 2.03 / 1.77 / −0.68  180 pA: t = 1.61 / 1.18 / −0.69 | Omnibus interaction:  Mafb cKO × current_pA: p = 0.600  c-Maf cKO × current_pA: p = 0.199  Holm–Šidák k = 27 (Mafb−WT / c-Maf−WT / c-Maf−Mafb):  20 pA: 1.000 / 1.000 / 1.000  40 pA: 1.000 / 1.000 / 1.000  60 pA: 1.000 / 0.999 / 1.000  80 pA: 1.000 / 0.999 / 0.998  100 pA: 1.000 / 0.999 / 0.997  120 pA: 1.000 / 1.000 / 0.992  140 pA: 1.000 / 1.000 / 1.000  160 pA: 0.809 / 0.925 / 1.000  180 pA: 0.999 / 1.000 / 1.000 | Omnibus interaction β (SE):  Mafb cKO × current_pA: 0.029 (0.055)  c-Maf cKO × current_pA: −0.076 (0.059)  Per-step:  20 pA: WT 5.4 ± 1.4; Mafb 5.0 ± 1.2; c-Maf 4.0 ± 1.2  40 pA: WT 13.5 ± 2.6; Mafb 13.8 ± 2.3; c-Maf 12.1 ± 2.5  60 pA: WT 25.6 ± 3.4; Mafb 24.5 ± 3.1; c-Maf 20.5 ± 3.6  80 pA: WT 35.4 ± 4.6; Mafb 36.0 ± 4.2; c-Maf 28.5 ± 4.4  100 pA: WT 42.4 ± 5.2; Mafb 43.3 ± 4.7; c-Maf 34.7 ± 4.4  120 pA: WT 47.7 ± 6.6; Mafb 53.8 ± 4.9; c-Maf 44.0 ± 4.5  140 pA: WT 37.2 ± 6.4; Mafb 46.5 ± 6.3; c-Maf 44.5 ± 3.4  160 pA: WT 35.0 ± 7.7; Mafb 53.8 ± 6.6; c-Maf 48.5 ± 3.7  180 pA: WT 36.3 ± 8.3; Mafb 59.0 ± 9.0; c-Maf 49.8 ± 5.9 |
| 4B | Action potential firing frequency vs current step, PV+ CINs | Per-step LMM (spike_count ~ Genotype + 1\|mouse_id); Holm–Šidák k = 27 across (3 contrasts × 9 current steps) within cell type. Omnibus interaction LMM additionally fit. | PV+ WT: 6 cells/3 mice  Mafb cKO: 17 cells/4 mice  c-Maf cKO: 34 cells/4 mice | Omnibus interaction:  Mafb cKO × current_pA: t(26.4) = −0.62  c-Maf cKO × current_pA: t(23.6) = −0.50  Per-step contrasts (Mafb−WT / c-Maf−WT / c-Maf−Mafb):  20 pA: t = 0.80 / 0.90 / 0.05  40 pA: t = 0.13 / 0.64 / 0.76  60 pA: t = −0.17 / 0.17 / 0.50  80 pA: t = −0.21 / 0.11 / 0.46  100 pA: t = −0.90 / −0.40 / 0.74  120 pA: t = −0.88 / −0.85 / 0.12  140 pA: t = −0.84 / −1.70 / −1.15  160 pA: t = −0.94 / −2.21 / −1.63  180 pA: t = −1.83 / −2.41 / −0.61 | Omnibus interaction:  Mafb cKO × current_pA: p = 0.539  c-Maf cKO × current_pA: p = 0.623  Holm–Šidák k = 27 (Mafb−WT / c-Maf−WT / c-Maf−Mafb):  20 pA: 1.000 / 1.000 / 1.000  40 pA: 1.000 / 1.000 / 1.000  60 pA: 1.000 / 1.000 / 1.000  80 pA: 1.000 / 1.000 / 1.000  100 pA: 1.000 / 1.000 / 1.000  120 pA: 1.000 / 1.000 / 1.000  140 pA: 1.000 / 0.922 / 0.999  160 pA: 1.000 / 0.754 / 0.987  180 pA: 0.937 / 0.835 / 1.000 | Omnibus interaction β (SE):  Mafb cKO × current_pA: −0.078 (0.125)  c-Maf cKO × current_pA: −0.059 (0.118)  Per-step:  20 pA: WT 0.0 ± 0.0; Mafb 2.6 ± 1.7; c-Maf 2.7 ± 1.2  40 pA: WT 2.7 ± 2.7; Mafb 7.8 ± 3.6; c-Maf 11.2 ± 2.7  60 pA: WT 4.3 ± 4.3; Mafb 18.0 ± 5.7; c-Maf 21.2 ± 3.8  80 pA: WT 16.0 ± 1.5; Mafb 28.5 ± 7.5; c-Maf 31.8 ± 4.9  100 pA: WT 33.0 ± 7.6; Mafb 37.5 ± 8.9; c-Maf 44.0 ± 5.1  120 pA: WT 45.7 ± 11.9; Mafb 51.0 ± 8.8; c-Maf 52.0 ± 5.1  140 pA: WT 55.3 ± 16.9; Mafb 63.0 ± 9.1; c-Maf 51.2 ± 5.4  160 pA: WT 65.7 ± 17.1; Mafb 73.5 ± 7.7; c-Maf 56.1 ± 6.6  180 pA: WT 79.3 ± 15.9; Mafb 65.8 ± 6.0; c-Maf 59.5 ± 7.2 |
| 4C | AP threshold (mV), SST+ CINs | Linear mixed-effects model (threshold ~ Genotype + 1\|mouse_id); Holm–Šidák k = 3 | SST+ WT: 26/3  Mafb cKO: 29/3  c-Maf cKO: 22/2 | Mafb − WT: t(3.62) = −0.83  c-Maf − WT: t(3.33) = 0.45  c-Maf − Mafb: t(3.32) = 1.25 | Mafb − WT: p = 0.706  c-Maf − WT: p = 0.706  c-Maf − Mafb: p = 0.646 | WT: −44.8 ± 0.9  Mafb cKO: −45.8 ± 0.8  c-Maf cKO: −44.0 ± 1.0 |
| 4C | AP amplitude (mV), SST+ CINs | Linear mixed-effects model (amplitude ~ Genotype + 1\|mouse_id); Holm–Šidák k = 3 | SST+ WT: 26/3  Mafb cKO: 29/3  c-Maf cKO: 22/2 | Mafb − WT: t(5.48) = 1.32  c-Maf − WT: t(5.07) = 1.93  c-Maf − Mafb: t(4.99) = 0.73 | Mafb − WT: p = 0.421  c-Maf − WT: p = 0.297  c-Maf − Mafb: p = 0.499 | WT: 41.9 ± 1.9  Mafb cKO: 46.5 ± 1.7  c-Maf cKO: 48.5 ± 1.5 |
| 4C | AP half-width (ms), SST+ CINs | Linear mixed-effects model (half_duration ~ Genotype + 1\|mouse_id); Holm–Šidák k = 3 | SST+ WT: 26/3  Mafb cKO: 29/3  c-Maf cKO: 22/2 | Mafb − WT: t(74) = −1.25  c-Maf − WT: t(74) = 0.26  c-Maf − Mafb: t(74) = 1.46 | Mafb − WT: p = 0.384  c-Maf − WT: p = 0.795  c-Maf − Mafb: p = 0.382 | WT: 1.04 ± 0.06  Mafb cKO: 0.95 ± 0.04  c-Maf cKO: 1.06 ± 0.05 |
| 4C | Rheobase (pA), SST+ CINs | Linear mixed-effects model (rheobase ~ Genotype + 1\|mouse_id); Holm–Šidák k = 3 | SST+ WT: 26/3  Mafb cKO: 29/3  c-Maf cKO: 22/2 | Mafb − WT: t(3.22) = −0.16  c-Maf − WT: t(2.97) = 0.46  c-Maf − Mafb: t(3.01) = 0.63 | Mafb − WT: p = 0.923  c-Maf − WT: p = 0.923  c-Maf − Mafb: p = 0.923 | WT: 40.0 ± 5.0  Mafb cKO: 38.6 ± 4.9  c-Maf cKO: 43.6 ± 6.0 |
| 4C | AP threshold (mV), PV+ CINs | Linear mixed-effects model (threshold ~ Genotype + Sex + 1\|mouse_id); Holm–Šidák k = 3 | PV+ WT: 6/3  Mafb cKO: 17/4  c-Maf cKO: 34/4 | Mafb − WT: t(9.36) = −0.12  c-Maf − WT: t(8.04) = −0.92  c-Maf − Mafb: t(5.35) = −0.94 | Mafb − WT: p = 0.910  c-Maf − WT: p = 0.766  c-Maf − Mafb: p = 0.766 | WT: −40.0 ± 2.1  Mafb cKO: −42.1 ± 1.3  c-Maf cKO: −43.0 ± 0.8 |
| 4C | AP amplitude (mV), PV+ CINs | Linear mixed-effects model (amplitude ~ Genotype + Sex + 1\|mouse_id); Holm–Šidák k = 3 | PV+ WT: 6/3  Mafb cKO: 17/4  c-Maf cKO: 34/4 | Mafb − WT: t(53) = 1.12  c-Maf − WT: t(53) = 2.07  c-Maf − Mafb: t(53) = 1.21 | Mafb − WT: p = 0.412  c-Maf − WT: p = 0.125  c-Maf − Mafb: p = 0.412 | WT: 32.7 ± 5.7  Mafb cKO: 37.5 ± 2.1  c-Maf cKO: 39.3 ± 1.2 |
| 4C | AP half-width (ms), PV+ CINs | Linear mixed-effects model (half_duration ~ Genotype + Sex + 1\|mouse_id); Holm–Šidák k = 3 | PV+ WT: 6/3  Mafb cKO: 17/4  c-Maf cKO: 34/4 | Mafb − WT: t(16.96) = −1.89  c-Maf − WT: t(15.07) = −1.22  c-Maf − Mafb: t(7.60) = 1.05 | Mafb − WT: p = 0.211  c-Maf − WT: p = 0.423  c-Maf − Mafb: p = 0.423 | WT: 0.94 ± 0.09  Mafb cKO: 0.69 ± 0.03  c-Maf cKO: 0.79 ± 0.04 |
| 4C | Rheobase (pA), PV+ CINs | Linear mixed-effects model (rheobase ~ Genotype + Sex + 1\|mouse_id); Holm–Šidák k = 3 (sex coefficient β = −25.0 pA, raw p = 0.021) | PV+ WT: 6/3  Mafb cKO: 17/4  c-Maf cKO: 34/4 | Mafb − WT: t(53) = 0.66  c-Maf − WT: t(53) = −0.30  c-Maf − Mafb: t(53) = −1.46 | Mafb − WT: p = 0.763  c-Maf − WT: p = 0.763  c-Maf − Mafb: p = 0.388 | WT: 63.3 ± 12.0  Mafb cKO: 67.0 ± 8.0  c-Maf cKO: 58.8 ± 5.7 |
| 4D | Resting Vm (mV), SST+ CINs | Linear mixed-effects model (Vm ~ Genotype + 1\|mouse_id); Holm–Šidák k = 3 | SST+ WT: 26/3  Mafb cKO: 29/3  c-Maf cKO: 22/2 | Mafb − WT: t(74) = 0.81  c-Maf − WT: t(74) = −1.71  c-Maf − Mafb: t(74) = −2.52 | Mafb − WT: p = 0.421  c-Maf − WT: p = 0.175  **c-Maf − Mafb: p = 0.041 *** | WT: −63.6 ± 0.7  Mafb cKO: −62.8 ± 0.5  c-Maf cKO: −65.3 ± 0.8 |
| 4D | Rin (MΩ), SST+ CINs | Linear mixed-effects model (Rin ~ Genotype + 1\|mouse_id); Holm–Šidák k = 3 | SST+ WT: 26/3  Mafb cKO: 29/3  c-Maf cKO: 22/2 | Mafb − WT: t(74) = −1.21  c-Maf − WT: t(74) = −0.59  c-Maf − Mafb: t(74) = 0.55 | Mafb − WT: p = 0.541  c-Maf − WT: p = 0.801  c-Maf − Mafb: p = 0.801 | WT: 592.3 ± 71.8  Mafb cKO: 496.6 ± 34.8  c-Maf cKO: 542.0 ± 65.4 |
| 4D | τm (ms), SST+ CINs | Linear mixed-effects model (tau_m ~ Genotype + 1\|mouse_id); Holm–Šidák k = 3 | SST+ WT: 26/3  Mafb cKO: 29/3  c-Maf cKO: 22/2 | Mafb − WT: t(74) = 0.28  c-Maf − WT: t(74) = −0.33  c-Maf − Mafb: t(74) = −0.61 | Mafb − WT: p = 0.934  c-Maf − WT: p = 0.934  c-Maf − Mafb: p = 0.907 | WT: 37.1 ± 4.6  Mafb cKO: 38.7 ± 4.0  c-Maf cKO: 35.0 ± 3.7 |
| 4D | Cm (pF), SST+ CINs | Linear mixed-effects model (Cm ~ Genotype + 1\|mouse_id); Holm–Šidák k = 3 | SST+ WT: 23/3 (3 cells lacked usable Cm)  Mafb cKO: 29/3  c-Maf cKO: 22/2 | Mafb − WT: t(71) = −1.57  c-Maf − WT: t(71) = 0.24  c-Maf − Mafb: t(71) = 1.81 | Mafb − WT: p = 0.226  c-Maf − WT: p = 0.810  c-Maf − Mafb: p = 0.208 | WT: 60.2 ± 11.3  Mafb cKO: 41.0 ± 5.5  c-Maf cKO: 63.3 ± 9.9 |
| 4D | Resting Vm (mV), PV+ CINs | Linear mixed-effects model (Vm ~ Genotype + Sex + 1\|mouse_id); Holm–Šidák k = 3 | PV+ WT: 6/3  Mafb cKO: 17/4  c-Maf cKO: 34/4 | Mafb − WT: t(53) = 0.00  c-Maf − WT: t(53) = 0.36  c-Maf − Mafb: t(53) = 0.51 | Mafb − WT: p = 1.00  c-Maf − WT: p = 0.943  c-Maf − Mafb: p = 0.943 | WT: −65.8 ± 1.2  Mafb cKO: −65.1 ± 1.0  c-Maf cKO: −65.0 ± 0.6 |
| 4D | Rin (MΩ), PV+ CINs | Linear mixed-effects model (Rin ~ Genotype + Sex + 1\|mouse_id); Holm–Šidák k = 3 | PV+ WT: 6/3  Mafb cKO: 17/4  c-Maf cKO: 34/4 | Mafb − WT: t(7.92) = −0.99  c-Maf − WT: t(6.92) = −0.27  c-Maf − Mafb: t(3.91) = 0.95 | Mafb − WT: p = 0.725  c-Maf − WT: p = 0.798  c-Maf − Mafb: p = 0.725 | WT: 404.2 ± 74.3  Mafb cKO: 326.5 ± 49.5  c-Maf cKO: 339.7 ± 27.1 |
| 4D | τm (ms), PV+ CINs | Linear mixed-effects model (tau_m ~ Genotype + Sex + 1\|mouse_id); Holm–Šidák k = 3 | PV+ WT: 6/3  Mafb cKO: 17/4  c-Maf cKO: 34/4 | Mafb − WT: t(12.20) = −1.83  c-Maf − WT: t(10.90) = −0.37  c-Maf − Mafb: t(4.22) = 2.03 | Mafb − WT: p = 0.250  c-Maf − WT: p = 0.716  c-Maf − Mafb: p = 0.250 | WT: 19.2 ± 3.3  Mafb cKO: 13.9 ± 1.5  c-Maf cKO: 17.2 ± 1.0 |
| 4D | Cm (pF), PV+ CINs | Linear mixed-effects model (Cm ~ Genotype + Sex + 1\|mouse_id); Holm–Šidák k = 3 | PV+ WT: 6/3  Mafb cKO: 17/4  c-Maf cKO: 34/4 | Mafb − WT: t(53) = −0.10  c-Maf − WT: t(53) = −1.12  c-Maf − Mafb: t(53) = −1.43 | Mafb − WT: p = 0.923  c-Maf − WT: p = 0.467  c-Maf − Mafb: p = 0.402 | WT: 78.3 ± 17.4  Mafb cKO: 66.5 ± 10.6  c-Maf cKO: 53.1 ± 9.7 |
| 5D | sEPSC amplitude (pA), SST+ CINs | Linear mixed-effects model (amplitude ~ Genotype + 1\|mouse_id); Holm–Šidák k = 3 | SST+ WT: 26/3  Mafb cKO: 29/3  c-Maf cKO: 22/2 | Mafb − WT: t(73) = −0.44  c-Maf − WT: t(73) = −0.39  c-Maf − Mafb: t(73) = 0.01 | Mafb − WT: p = 0.962  c-Maf − WT: p = 0.962  c-Maf − Mafb: p = 0.989 | WT: 17.1 ± 1.2  Mafb cKO: 16.6 ± 0.6  c-Maf cKO: 16.6 ± 0.5 |
| 5D | sEPSC decay tau (ms), SST+ CINs | Linear mixed-effects model (decay_tau ~ Genotype + 1\|mouse_id); Holm–Šidák k = 3 | SST+ WT: 26/3  Mafb cKO: 29/3  c-Maf cKO: 22/2 | Mafb − WT: t(73) = 0.49  c-Maf − WT: t(73) = 1.54  c-Maf − Mafb: t(73) = 1.14 | Mafb − WT: p = 0.625  c-Maf − WT: p = 0.338  c-Maf − Mafb: p = 0.450 | WT: 3.07 ± 0.35  Mafb cKO: 3.31 ± 0.32  c-Maf cKO: 3.87 ± 0.39 |
| 5D | sEPSC charge (fC), SST+ CINs | Linear mixed-effects model (charge ~ Genotype + 1\|mouse_id); Holm–Šidák k = 3 | SST+ WT: 26/3  Mafb cKO: 29/3  c-Maf cKO: 22/2 | Mafb − WT: t(73) = 0.91  c-Maf − WT: t(73) = 1.32  c-Maf − Mafb: t(73) = 0.50 | Mafb − WT: p = 0.598  c-Maf − WT: p = 0.473  c-Maf − Mafb: p = 0.621 | WT: 64.0 ± 5.7  Mafb cKO: 71.3 ± 5.7  c-Maf cKO: 75.4 ± 5.8 |
| 5E | sEPSC instantaneous frequency (Hz), SST+ CINs | Linear mixed-effects model (inst_freq ~ Genotype + 1\|mouse_id); Holm–Šidák k = 3. Mouse-level confirmation: one-way ANOVA + Tukey HSD on per-mouse means. | SST+ WT: 26/3  Mafb cKO: 29/3  c-Maf cKO: 22/2 | Mafb − WT: t(73) = 0.39  c-Maf − WT: t(73) = −2.74  c-Maf − Mafb: t(73) = −3.25  Mouse-level ANOVA: F(2, 5) = 58.79 | Mafb − WT: p = 0.701  **c-Maf − WT: p = 0.016 ***  **c-Maf − Mafb: p = 0.005 ****  Mouse-level Tukey:  **c-Maf − WT: p = 7 × 10⁻⁴ *****  **c-Maf − Mafb: p = 4 × 10⁻⁴ ***** | WT: 4.99 ± 0.96  Mafb cKO: 5.47 ± 1.02  c-Maf cKO: 1.38 ± 0.31 |
| 5D | sEPSC amplitude (pA), PV+ CINs | Linear mixed-effects model (amplitude ~ Genotype + Sex + 1\|mouse_id); Holm–Šidák k = 3 | PV+ WT: 6/3  Mafb cKO: 17/4  c-Maf cKO: 34/4 | Mafb − WT: t(8.46) = 0.88  c-Maf − WT: t(7.71) = 1.28  c-Maf − Mafb: t(4.60) = 0.44 | Mafb − WT: p = 0.644  c-Maf − WT: p = 0.560  c-Maf − Mafb: p = 0.680 | WT: 14.1 ± 1.1  Mafb cKO: 16.4 ± 0.9  c-Maf cKO: 16.8 ± 0.5 |
| 5D | sEPSC decay tau (ms), PV+ CINs | Linear mixed-effects model (decay_tau ~ Genotype + Sex + 1\|mouse_id); Holm–Šidák k = 3 | PV+ WT: 6/3  Mafb cKO: 17/4  c-Maf cKO: 34/4 | Mafb − WT: t(28.76) = −1.14  c-Maf − WT: t(26.63) = −0.26  c-Maf − Mafb: t(11.90) = 1.31 | Mafb − WT: p = 0.519  c-Maf − WT: p = 0.800  c-Maf − Mafb: p = 0.519 | WT: 1.94 ± 0.34  Mafb cKO: 1.15 ± 0.14  c-Maf cKO: 1.93 ± 0.29 |
| 5D | sEPSC charge (fC), PV+ CINs | Linear mixed-effects model (charge ~ Genotype + Sex + 1\|mouse_id); Holm–Šidák k = 3 | PV+ WT: 6/3  Mafb cKO: 17/4  c-Maf cKO: 34/4 | Mafb − WT: t(52) = −0.66  c-Maf − WT: t(52) = 0.37  c-Maf − Mafb: t(52) = 1.51 | Mafb − WT: p = 0.762  c-Maf − WT: p = 0.762  c-Maf − Mafb: p = 0.356 | WT: 33.1 ± 6.6  Mafb cKO: 26.4 ± 2.9  c-Maf cKO: 39.5 ± 4.3 |
| 5E | sEPSC instantaneous frequency (Hz), PV+ CINs | Linear mixed-effects model (inst_freq ~ Genotype + Sex + 1\|mouse_id); Holm–Šidák k = 3 | PV+ WT: 6/3  Mafb cKO: 17/4  c-Maf cKO: 34/4 | Mafb − WT: t(11.87) = 0.46  c-Maf − WT: t(11.06) = 1.50  c-Maf − Mafb: t(7.80) = 1.20 | Mafb − WT: p = 0.655  c-Maf − WT: p = 0.411  c-Maf − Mafb: p = 0.462 | WT: 5.46 ± 1.71  Mafb cKO: 8.09 ± 1.35  c-Maf cKO: 13.36 ± 1.67 |
| 5-1B/C | Dendritic synaptic puncta density (puncta/pixel), SST+ CINs, 2 mo | Primary: pairwise likelihood-ratio tests on lognormal fits at dendrite level, Bonferroni m = 3.  Confirmatory: LMM (puncta/pixel ~ Genotype + 1\|mouse_id), Holm–Šidák k = 3.  Confirmatory: one-way ANOVA + Tukey HSD on per-mouse means. | 10 dendrites/genotype from 2 mice/genotype (1 M + 1 F per group); 30 dendrites / 6 mice total | LRT:  WT vs Mafb: χ²(2) = 2.20  WT vs c-Maf: χ²(2) = 10.96  Mafb vs c-Maf: χ²(2) = 4.62  LMM:  Mafb − WT: t(2.87) = −1.09  c-Maf − WT: t(2.87) = −2.19  c-Maf − Mafb: t(2.87) = −1.10  Mouse-level ANOVA:  F(2, 3) = 2.49  Tukey HSD (mean diff, 95% CI):  Mafb − WT: −0.074 [−0.324, 0.175]  c-Maf − WT: −0.133 [−0.382, 0.116]  c-Maf − Mafb: −0.058 [−0.308, 0.191] | LRT (Bonferroni):  WT vs Mafb: p = 1.00  **WT vs c-Maf: p = 0.013 ***  Mafb vs c-Maf: p = 0.297  LMM (Holm–Šidák):  Mafb − WT: p = 0.586  c-Maf − WT: p = 0.319  c-Maf − Mafb: p = 0.586  Mouse-level ANOVA:  p = 0.23  Tukey HSD:  Mafb − WT: p = 0.509  c-Maf − WT: p = 0.212  c-Maf − Mafb: p = 0.636 | Dendrite-level mean ± SEM  WT: 0.290 ± 0.033  Mafb cKO: 0.234 ± 0.029  c-Maf cKO: 0.163 ± 0.032  Dendrite-level median:  WT: 0.275  Mafb cKO: 0.224  c-Maf cKO: 0.143  Mouse-level mean ± SEM  WT: 0.294 ± 0.021  Mafb cKO: 0.220 ± 0.070  c-Maf cKO: 0.161 ± 0.007 |
