# Supplementary figures and images for: c-Maf Deletion in Cortical Somatostatin, But Not Parvalbumin, Interneurons Leads to Absence-Like Epileptiform Activity in Mice
Source: eNeuro. 2026 Jul 17;13(7):ENEURO.0257-25.2026. doi: 10.1523/ENEURO.0257-25.2026 (PMC13395531; doi:10.1523/ENEURO.0257-25.2026)

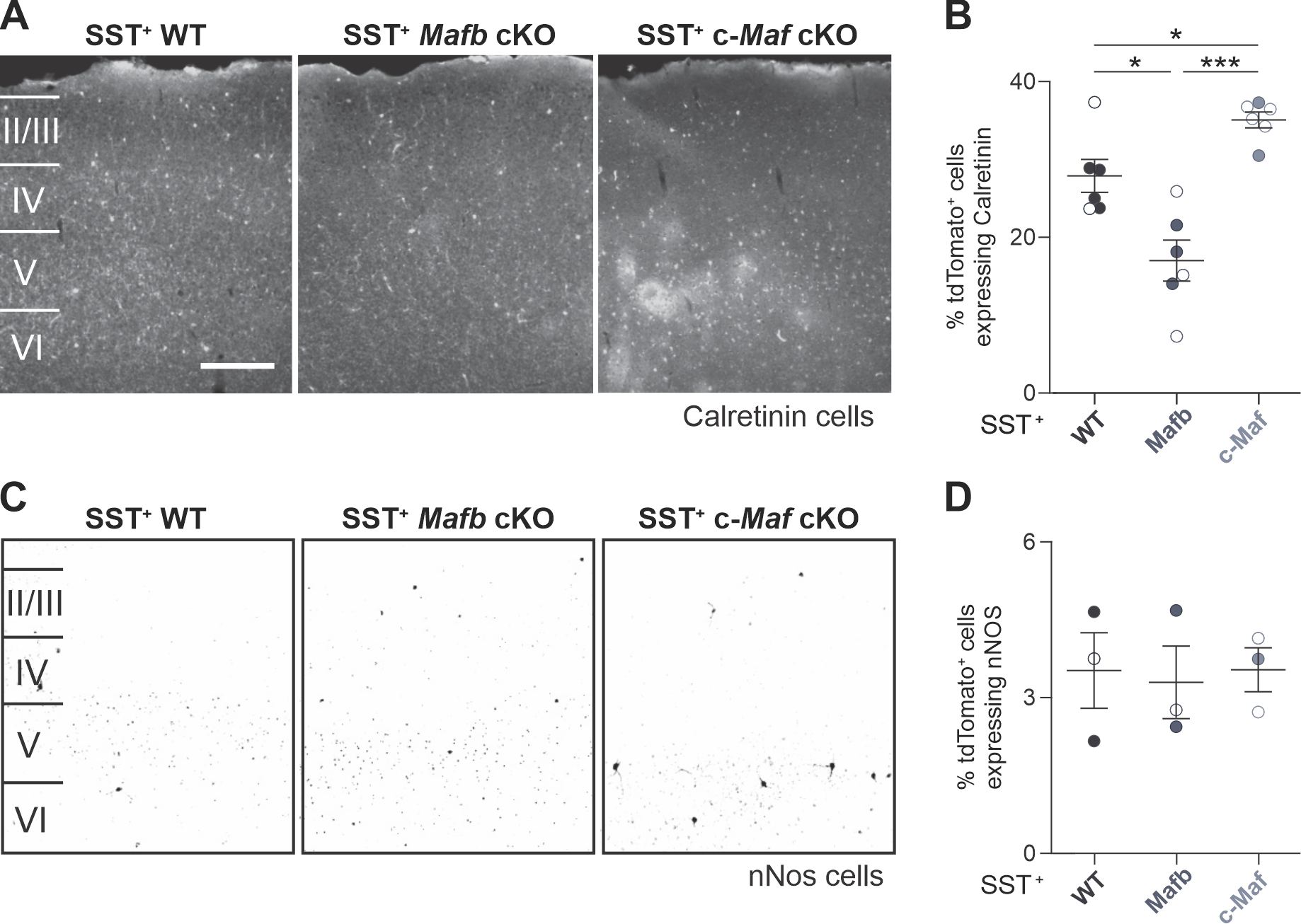

Supplement: Figure 3-1 — Effect of Mafb or c-Maf deletion on SST+ subtype numbers in S1 cortex. A, Immunofluorescent images from WT, SST+ Mafb cKO, and SST+ c-Maf cKO mice showing Calretinin + CINs (8–10-month-old mice). B, Quantification of Martinotti cells (double positive for tdTomato+ driven by SST-Cre and Calretinin). These results were obtained from mice that were age-matched with those subjected to in vivo ECoG recordings in Figure 1. n = 6 mice per genotype including both male and female mice (WT: n = 4 males, 2 females; SST+ Mafb cKO: n = 3 males, 3 females; SST+ c-Maf cKO: n = 2 males, 4 females). C, Immunofluorescent images showing immunolabeled nNOS+ CINs. D, Quantification of long-range SST+ projection neurons (double positive for tdTomato+ driven by SST-Cre and nNOS) in 8–10-month-old mice. n = 3 mice per genotype including both male and female mice. WT: n = 2 males, 1 female; SST+ Mafb cKO: n = 2 males, 1 female; SST+ c-Maf cKO: n = 1 male, 2 females. Statistical significance was assessed by one-way ANOVA followed by pairwise t-tests with Holm–Šidák correction (k = 3 pairwise genotype contrasts) at the all-layers level; parametric assumptions verified by Shapiro–Wilk and Levene tests (***p < 0.001; **p < 0.01; *p < 0.05). Males are represented by gray dots and females by white dots. Scale bars: 200 μm. See Figure 1-1 for complete statistical reporting. Download Figure 3-1, TIF file. [file eneuro-13-ENEURO.0257-25.2026-s002.tif]

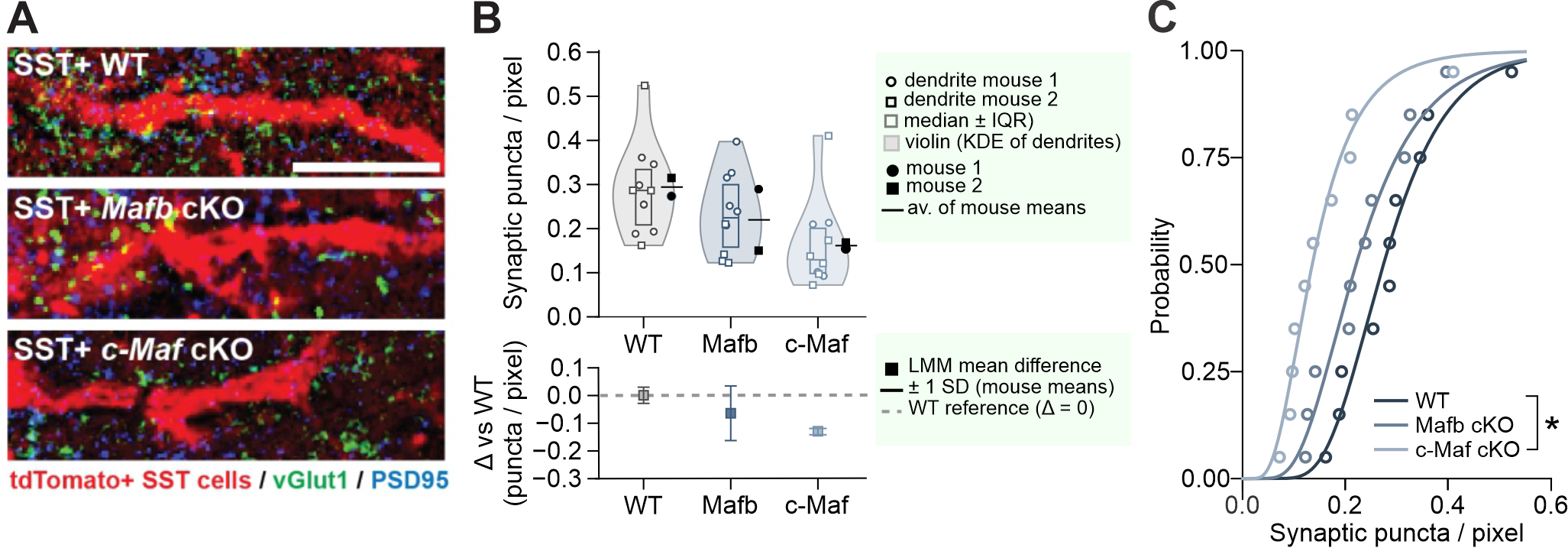

Supplement: Figure 5-1 — Exploratory analysis of the effect of Mafb or c-Maf deletion in SST+ CINs on the density of excitatory synapses on their proximal dendrites in adult mice. A, Immunofluorescent images from the S1 cortex of WT, SST+ Mafb cKO, and SST+ c-Maf cKO mice. CINs are marked with tdTomato and excitatory synapses are marked with vGlut1 and PSD95. B, Synapse density (puncta per pixel) on tdTomato + dendrites of SST + CINs in S1 cortex, shown as overlaid violin (kernel density), box (median and IQR), and individual dendrite measurements. Open symbols are individual dendrites color-coded by mouse of origin (circle = mouse 1, square = mouse 2); filled symbols are the mean of each mouse's dendrites; horizontal line is the average of the two mouse means per genotype. Lower sub-panel (Δ vs WT): mean difference relative to WT estimated by linear mixed-effects regression, with error bars showing ± 1 SD of the per-mouse means within each cKO group; dashed gray line marks the WT reference (Δ = 0). C, Empirical cumulative distribution functions (circles) and maximum-likelihood lognormal fits (solid lines) of dendritic synaptic puncta density per genotype. The c-Maf cKO distribution shows a ∼48% reduction in median puncta density relative to WT (0.143 vs 0.275 puncta/pixel). Statistical significance was assessed by pairwise likelihood-ratio tests on lognormal fits at the dendrite level (Bonferroni-corrected) and confirmed by linear mixed-effects regression and one-way ANOVA on per-mouse means; only the dendrite-level test reached significance for c-Maf cKO vs WT (*p = 0.013); all other contrasts were ns (*p < 0.05; ns, not significant). See Methods for details. Synaptic puncta analyses were performed in two-month-old mice, consistent with the age used for patch-clamp electrophysiology experiments in Figures 4 and 5. n = 10 dendrites from 2 mice (one male, one female) per genotype (30 dendrites from 6 mice total). Scale bar: 5 μm. See Figure 1-1 for complete statistical reporting. Download F [file eneuro-13-ENEURO.0257-25.2026-s003.tif]
